# Drug Repositioning of Inflammatory Bowel Disease Based on Co-Target Gene Expression Signature of Glucocorticoid Receptor and TET2

**DOI:** 10.3390/biology13020082

**Published:** 2024-01-29

**Authors:** Xianglin Zhao, Chenghao Hu, Xinyu Chen, Shuqiang Ren, Fei Gao

**Affiliations:** 1 Genome Analysis Laboratory of the Ministry of Agriculture and Rural Affairs, Agricultural Genomics Institute at Shenzhen, Chinese Academy of Agricultural Sciences, Shenzhen 518000, China; 2 School of Life Sciences, Henan University, Kaifeng 475004, China; 3 Shenzhen Research Institute of Henan University, Henan University, Shenzhen 518000, China; 4 College of Animal Science and Technology, Guangxi University, Nanning 530004, China; 5 HIM-BGI Omics Center, Zhejiang Cancer Hospital, Hangzhou Institute of Medicine (HIM), Chinese Academy of Sciences (CAS), Hangzhou 310022, China; 6 Comparative Pediatrics and Nutrition, Department of Veterinary and Animal Sciences, Faculty of Health and Medical Sciences, University of Copenhagen, DK-2100 Copenhagen, Denmark

**Keywords:** IBD, GR, TET2, drug repositioning, in vitro inflammatory model

## Abstract

**Simple Summary:**

Inflammatory bowel disease (IBD), encompassing ulcerative colitis (UC) and Crohn’s disease (CD), impacts millions globally and is characterized by complex immune responses. This research aimed to unravel the collaborative roles of two critical protein factors in immune regulation within IBD. Through extensive analysis of public datasets, we identified specific gene signatures associated with these factors in IBD. Subsequent drug repositioning efforts and the utilization of cellular models led to the identification of BMS-536924 as a promising anti-inflammatory agent. This investigation has enriched our understanding of IBD’s pathogenesis and suggests novel therapeutic approaches to enhance the quality of life for individuals affected by these inflammatory conditions.

**Abstract:**

The glucocorticoid receptor (GR) and ten-eleven translocation 2 (TET2), respectively, play a crucial role in regulating immunity and inflammation, and GR interacts with TET2. However, their synergetic roles in inflammatory bowel disease (IBD), including ulcerative colitis (UC) and Crohn’s disease (CD), remain unclear. This study aimed to investigate the co-target gene signatures of GR and TET2 in IBD and provide potential therapeutic interventions for IBD. By integrating public data, we identified 179 GR- and TET2-targeted differentially expressed genes (DEGs) in CD and 401 in UC. These genes were found to be closely associated with immunometabolism, inflammatory responses, and cell stress pathways. In vitro inflammatory cellular models were constructed using LPS-treated HT29 and HCT116 cells, respectively. Drug repositioning based on the co-target gene signatures of GR and TET2 derived from transcriptomic data of UC, CD, and the in vitro model was performed using the Connectivity Map (CMap). BMS-536924 emerged as a top therapeutic candidate, and its validation experiment within the in vitro inflammatory model confirmed its efficacy in mitigating the LPS-induced inflammatory response. This study sheds light on the pathogenesis of IBD from a new perspective and may accelerate the development of novel therapeutic agents for inflammatory diseases including IBD.

## 1. Introduction

Inflammatory bowel disease (IBD), primarily comprising ulcerative colitis (UC) and Crohn’s disease (CD), is a chronic and relapsing inflammatory disorder of the intestine that profoundly affects patients’ quality of life. Although previous research efforts suggest that the disease is caused by several factors, including genetic and epigenomic influences, alterations in intestinal microbiota, and mucosal immune imbalance, its pathogenesis is not yet fully understood [1,2,3]. With the increasing prevalence and complexity of IBD [4,5], there is an urgent need to achieve deeper disease control, innovate therapeutic strategies, and personalize care. Recently, the establishment of specific gene expression signatures for screening promising drug candidates with the Connectivity Map (CMap) and developing novel therapies has been applied rapidly and efficiently [6,7,8].

The glucocorticoid receptor (GR), a nuclear receptor present in nearly all tissues, exhibits potent immunomodulatory and anti-inflammatory effects. Its action chiefly involves suppressing proinflammatory transcription factors such as AP-1 and NF-κB, modulating anti-inflammatory genes, and inducing lymphocyte apoptosis [9,10,11]. Recent studies have unveiled GR’s critical role in intestinal epithelial cells, particularly in controlling colonic inflammation by regulating the gene expression of chemokines, leukocyte recruitment, and epithelial barrier permeability [12]. Moreover, there is growing evidence about the significance of epigenetic regulation by TET2, a ten-eleven translocation (TET) family enzyme catalyzing the oxidation of 5mC to 5hmC, in maintaining innate immune balance and resolving inflammation [13,14]. For instance, TET2 deficiency results in macrophages exhibiting an increase in NLRP3 inflammasome-mediated IL-1β secretion, which aggravates the inflammation phenotype [15]. TET2 also plays a role in inflammation resolution by repressing the expression of the inflammatory cytokine IL-6 expression in innate myeloid cells, including dendritic cells and macrophages, through histone deacetylation facilitated by HDAC2 recruitment, a process initiated by the IL-1R-MyD88 pathway [16]. The interaction between GR and TET2, mediated by MAFB in tolerogenic dendritic cells (tolDCs), has been demonstrated [17], and our group confirmed this interaction in HEK293T cells (unpublished data). This evidence indicates that genes co-regulated by GR and TET2 may significantly correlate with inflammatory response. However, little is known about their specific roles and mechanisms in IBD.

In this study, we performed a bioinformatics analysis aimed at assessing the role of GR and TET2 regulation in IBD and elucidating the molecular mechanisms underlying the regulation of downstream genes by GR and TET2 in the disease, along with its potential implications for drug repositioning. Meanwhile, holistic gene expression programs on the LPS-stimulated cells with different GR baseline levels, i.e., HT29 and HCT116 cells, were compared. Furthermore, anti-inflammatory small-molecular compounds were identified after comprehensively applying multiple gene signatures associated with GR and TET2, and the top-ranked compound was validated in an inflammation-sensitive HT29 model. This work has helped deepen the understanding of IBD pathogenesis and provides a new perspective on therapeutic strategies for IBD.

## 2. Materials and Methods

### 2.1. Collection and Pre-Processing of Public Transcriptomic Datasets

To investigate the tissue transcriptome of IBD, we utilized NCBI’s Gene Expression Omnibus (GEO) as our primary data source. Recognizing that sample location within IBD would have a large effect on the disease’s expression patterns [18,19,20], we selectively analyzed colon samples from untreated patients with active disease and healthy controls from seven microarray experiments. The details of these datasets are shown in Table S1. The sample size comprised 227 UC samples, 54 CD samples, and 84 healthy control (HC) samples. Quality control was performed on each gene expression dataset via background adjustment, quantile normalization, log2 transformation, and conversion of probes to gene symbols. Subsequently, we integrated these datasets and corrected for batch effects using the R package “sva” [21] (version 3.46.0).

### 2.2. Acquisition of GR and TET2 Putative Co-Target Genes (GTPCGs)

The Cistrome Data Browser (CistromeDB, http://cistrome.org/db (accessed on 26 June 2023)) serves as a comprehensive database, curating and analyzing a plethora of chromatin profiling assays, including ChIP-seq, DNase-seq, and ATAC-seq [22]. For our study, we selected high-quality peak files of GR and TET2 obtained from ChIP-seq data in the HEK293T cell line, a widely used model in scientific research. These files, sourced from CistromeDB (accession numbers: 41735, 33992, 67858), were then processed for further analysis. The binding peaks in each file were annotated to their corresponding genes using GREAT [23] (version 4.0.4, http://great.stanford.edu/ (accessed on 28 June 2023)) with default parameters, and a cross-analysis of each gene set was conducted, resulting in the identification of GR and TET2 putative co-regulated genes, referred to as GTPCGs. The complete list of these genes, totaling 6101, is detailed in Table S2.

### 2.3. Assessment of the Role of GR and TET2 in IBD

We meticulously collected 17 IBD biomarkers from recent literature, applying stringent criteria that necessitated their identification through both bioinformatics analysis and experimental validation [19,24,25,26,27,28,29]. Pearson’s correlation analysis was conducted to explore the association between *NR3C1* (which encodes GR) and *TET2* expression with these biomarkers. Based on the principle of linear support vector regression, a computational approach, i.e., cell-type identification by estimating relative subsets of RNA scripts (CIBERSORT), was leveraged to estimate the relative percentage of immune cell infiltration in bulk tissue transcriptome profiles [30,31]. Subsequently, we performed Spearman’s correlation analysis to investigate the relationship between immune cells, identified as significantly varied between IBD and HC, and four markers that intersected with both the 17 markers and GTPCGs. Additionally, ROC analysis was carried out to evaluate the diagnostic efficacy of these four markers for IBD using the “pROC” (version 1.18.0) and “rms” (version 6.7.0) R packages.

### 2.4. Differential Expression Gene Analysis

Differential analysis of microarray data in UC/HC and CD/HC comparisons was performed using the R package “limma” [32] (version 3.54.2). Differentially expressed genes (DEGs) were identified with the thresholds of adjust *p*-value < 0.05 and |log2 FC| > 0.58. We then compared the expression patterns of DEGs associated with UC and CD.

### 2.5. Identification of Specific Gene Signatures in UC and CD and Functional Enrichment Analysis

Specific gene signatures indicative of the disease effects in vivo were obtained by overlapping UC and CD DEGs with GTPCGs, respectively. Functional enrichment analyses were performed based on the Gene Ontology (GO) database and the Molecular Signatures database (MSigDB) using the R packages “clusterProfiler” (version 4.6.2), “org.Hs.eg.db” (version 3.16.0) and “enrichR” (version 3.2) [33,34]. To be specific, GO terms, including molecular function (MF), cellular component (CC), biological process (BP), and MSigDB hallmark pathways were enriched with the threshold of a *q*-value < 0.05. Redundant GO terms were removed based on semantic similarity using the “rrvgo” (version 1.10.0) R package. Ultimately, the top 10 most significant terms or pathways were revealed and visualized.

### 2.6. Cell-Type-Specific Enrichment Analysis

The cell-type-specific Enrichment Analysis database (CSEA-DB, https://bioinfo.uth.edu/CSEADB/ (accessed on 3 July 2023)) was used to investigate which cell type could be responsible for the signatures observed [35,36]. Among 126 general cell types from 111 tissues, we focused on cell types present in the intestine. This focus resulted in 7 tissues and 23 general cell types, which were identified through multiple testing corrections (BH method, FDR < 0.05) and filtering for cell types with a frequency of occurrence greater than or equal to 10 times.

### 2.7. Cell Culture and In Vitro Inflammatory Model

The HT29 [37,38] and HCT116 [39,40] cell lines were sourced from the American Type Culture Collection (ATCC). These cell lines were cultured in Advanced DMEM/F-12 medium (Gibco, Grand Island, NY, USA), supplemented with 10% fetal bovine serum (Sigma, St. Louis, NI, USA), 1% GlutaMAX Supplement (Gibco, Grand Island, NY, USA), and 1% TransSafe Mycoplasma Prevention Reagent (TRANS, Beijing, China). The culture conditions were maintained at 37 °C in a 5% CO_2_ atmosphere. To establish an in vitro inflammatory model of intestinal epithelial cells, the cells were treated with lipopolysaccharide (LPS, 1 ug/mL) (Sigma–Aldrich, St. Louis, MI, USA) for a duration of 24 h.

### 2.8. RNA-Seq Library Construction, Sequencing, and Data Analysis

In brief, total RNA was used as input material for the RNA sample preparations. Sequencing libraries were constructed using the NEBNext Ultra RNA Library Prep Kit for Illumina (NEB, Ipswich, MA, USA) according to the manufacturer’s instructions and then quantified using the Agilent 5400 system (Agilent, Santa Clara, CA, USA) and qPCR. The qualified cDNA libraries were sequenced with 150 bp paired-end reads on the Illumina NovaSeq 6000 platform (Illumina, San Diego, CA, USA). Raw transcriptome reads were cleaned and then aligned to the reference genome (hg38) using HISAT2 [41] (version 2.1.0). The annotated gene information was downloaded from GENCODE. A gene count matrix was generated by featureCounts [42] (version 2.0.1), and the “edgeR” (version 3.40.2) package was used for differential expression analysis [43].

### 2.9. Gene Set Enrichment Analysis

To explore the overarching gene expression patterns and identify the biological pathways predominantly active during LPS-induced inflammation in HT29 and HCT116 cells, we initially sorted genes in descending order based on their log2 fold change (FC) generated by edgeR. We then performed gene set enrichment analysis (GSEA) with the gseKEGG function from the “clusterProfiler” R package. The results with a cutoff criterion of a nominal *p*-value < 0.05 were considered statistically significant.

### 2.10. Rank-Rank Hypergeometric Overlap (RRHO) Analysis and Acquisition of Distinct Gene Signatures from the In Vitro Inflammation Model

Rank-rank hypergeometric overlap (RRHO) analysis is a threshold-free and robust approach to compare differential expression (DE) patterns between two experimental groups [44,45]. For HT29 and HCT116 cell groups subjected to LPS exposure, genes detected in both profiling experiments were ranked by their *p*-value and direction of effect size. Ranked lists were then compared to identify significantly overlapping genes across a continuous significance gradient by iterating the hypergeometric test. The results were presented in a heatmap using the “RRHO2” (version 1.0) R package [45]. We next identified a specific gene signature consisting of 75 genes that represent simulated disease effects common to both cell models. This signature was derived by intersecting GTPCGs with 407 genes, which were the overlapping genes obtained from the up-regulated gene lists, exhibiting the most significant overlap between the two cell groups. To reflect the biological or disease response of this gene signature, Kyoto Encyclopedia of Genes and Genomes (KEGG) pathway enrichment analysis was performed. A threshold of *p* < 0.05 was regarded to be significant enrichment.

### 2.11. Connectivity Map (CMap) Analysis

The Connectivity Map (CMap, http://clue.io/ (accessed on 2 July 2023)), an online analysis platform that conducts pattern-matching algorithms to compare query signatures with expression profiles in diverse contexts of over 450,000 chemical perturbagens, was leveraged to identify anti-inflammatory small molecules that create gene expression patterns opposite to the disease effect [6,7]. We submitted the three gene signatures previously obtained for CMap analysis, respectively. Each list of matched results was filtered with a cutoff of normalized connectivity score < −1 and cross-analyzed to obtain overlapping compounds. We next calculated their rankings within each list and determined the median, ultimately identifying the top 10 potentially anti-inflammatory agents. The chemical structures of selected molecules were collected from ChemSpider (https://chemspider.com (accessed on 9 September 2023)) [46].

### 2.12. Cell Viability Assay and In Vitro Treatment with Candidate Compounds

To determine the effect of candidate compounds on cell viability, the Cell Counting Kit-8 (CCK8) assay was used following the manufacture’s protocol. Initially, HT29 cells were plated in 96-well plates at a density of 1 × 10^4^ cells per well. After being cultured in the medium for 24 h, the cells were treated with different concentrations of BMS-536924 solution (ranging from 10 to 500 nM) for another 24 h. Subsequently, 10 uL CCK8 solution was added to each well, followed by incubation for 1 h at 37 °C. Cell viability was detected using a microplate spectrophotometer (Tecan Spark, Männedorf, Switzerland) at an optical density (OD) of 450 nm. For the compound treatment experiment, HT29 cells were seeded in 12-well plates at 1.5 × 10^5^ cells per well. These cells were treated with either LPS or medium for 24 h, followed by an additional 24 h incubation with BMS-536924 solution (at concentrations of 5, 10, and 50 nM) or medium. Cells treated solely with medium served as negative controls (NC group), and those only with LPS served as positive controls (LPS group).

### 2.13. Quantitative Real-Time Polymerase Chain Reaction (qRT-PCR)

Total RNA was extracted from the cells using the RNeasy Mini Kit (Qiagen, Duesseldorf, Germany). Reverse transcription was performed to synthesize cDNA with the PrimeScriptTM RT reagent Kit (Takara, Tokyo, Japan). The expression levels of specific genes were analyzed by a quantitative real-time PCR system (Thermo, Waltham, MA, USA) with the PowerUp SYBR Green Master Mix (Applied Biosystems, Foster City, CA, USA). Relative expression of each gene was normalized to GAPDH. The value of the control group was set to 1. qRT-PCR primers are listed in Table 1.

### 2.14. Statistical Analysis

All statistical analyses were conducted using R software (version 4.2.2) and GraphPad Prism (version 8.0.2). Results are presented as mean ± standard deviation (SD). Group differences were compared using an unpaired Student’s *t*-test, and statistical significance was defined as *p* < 0.05.

## 3. Results

### 3.1. Implications of GR and TET2 Regulation in IBD

**Figure 1:**
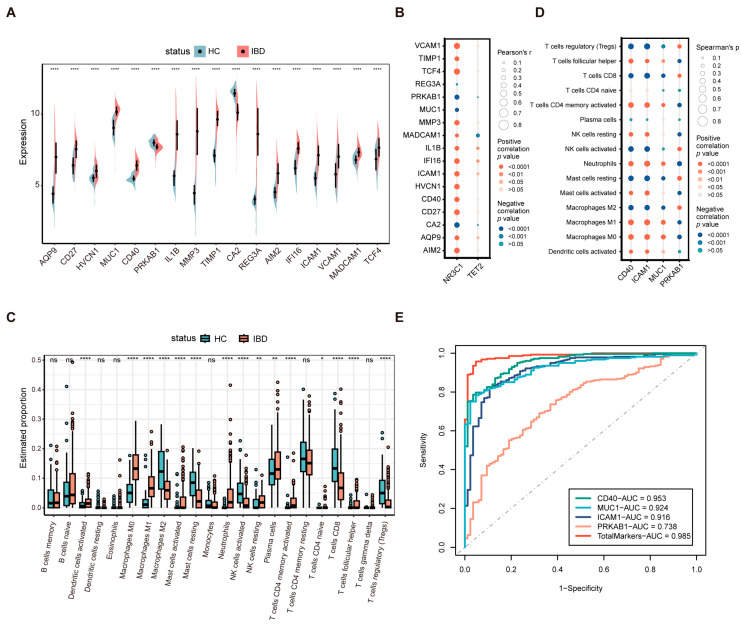
Assessment of the role of GR and TET2 regulation in IBD. (**A**) Split violin plot revealing the expressional differences of the 17 biomarkers between IBD and HC. (**B**) Correlating GR and TET2 separately with the 17 markers in mRNA level. (**C**) Box plot showing the comparison of 22 kinds of immune cells between IBD and HC. (**D**) Correlating the differentially infiltrated immune cells with the four markers upon the threshold of *p* < 0.05. (**E**) ROC curves of the four markers. * *p* < 0.05; ** *p* < 0.01; **** *p* < 0.0001; ns, not significant.

We extensively collected public transcriptomes of colon tissue in IBD, finally selecting datasets GSE75214, GSE16879, GSE179285, GSE36807, GSE73661, GSE9452, and GSE13367 from the GEO database (Table S1). After correcting and integrating the microarray datasets for batch effects, the PCA plots as well as the heatmap confirmed the elimination of batch effects among the datasets (Figure S1A–C). To investigate the relationship between GR and TET2 modulation and IBD, we collected literature-based IBD biomarkers and evaluated the expression of *NR3C1* and *TET2* in relation to these markers. Seventeen refined IBD markers from prior research were selected [19,24,25,26,27,28,29]. The split violin plot revealed significant expressional differences between IBD patients and HC (Figure 1A). Both significantly up-regulated and down-regulated genes showed expression trends consistent with those reported in their studies, suggesting that these markers were well validated in our dataset. The mRNA-level correlations between *NR3C1*, *TET2*, and these markers were examined. Except for *REG3A*, *NR3C1* was significantly associated with all markers, while *TET2* was significantly correlated with 5 of the 17 markers, including *AQP9*, *IL1B*, *IFI16*, *ICAM1*, and *MADCAM1* (Figure 1B).

Given that immune dysregulation is one of the pathogenic mechanisms of IBD, the CIBERSORT algorithm was employed to characterize the abundance of 22 immune cell infiltrates in colon tissue derived from IBD patients and HC. Figure 1C illustrates that multiple immune cell subpopulations were significantly altered between groups. Compared with HC, patients with IBD showed higher proportions of activated dendritic cells, M0 and M1 macrophages, activated mast cells, neutrophils, resting NK cells, plasma cells, activated CD4 memory T cells, and follicular helper T cells and lower proportions of M2 macrophages, resting mast cells, activated NK cells, CD8 T cells, and regulatory T cells (Tregs). The association between the expression of the four biomarkers (*CD40*, *ICAM1*, *MUC1*, and *PRKAB1*) and the proportion of immune cell types showing significant differences was further explored. As shown in Figure 1D, activated dendritic cells, M0 and M1 macrophages, activated mast cells, neutrophils, resting NK cells, CD4 memory-activated T cells, and follicular helper T cells exhibited positive correlations with *CD40*, *ICAM1*, and *MUC1* while displaying negative correlations with *PRKAB1*. Meanwhile, M2 macrophages, resting mast cells, activated NK cells, CD8 T cells, and Tregs presented a negative correlation with *CD40*, *ICAM1*, and *MUC1* while showing a positive correlation with *PRKAB1*. Moreover, we performed an ROC analysis to verify the diagnostic significance of the four markers. The markers had area under the curve (AUC) values > 0.7, with the total combination of them exhibiting the largest AUC value (AUC, 0.985) and *PRKAB1* the smallest (AUC, 0.738) (Figure 1E). Taken together, these collective results indicate that GR and TET2 regulation is likely to play a crucial role in IBD.

### 3.2. Identification of DEGs Associated with UC and CD

**Figure 2:**
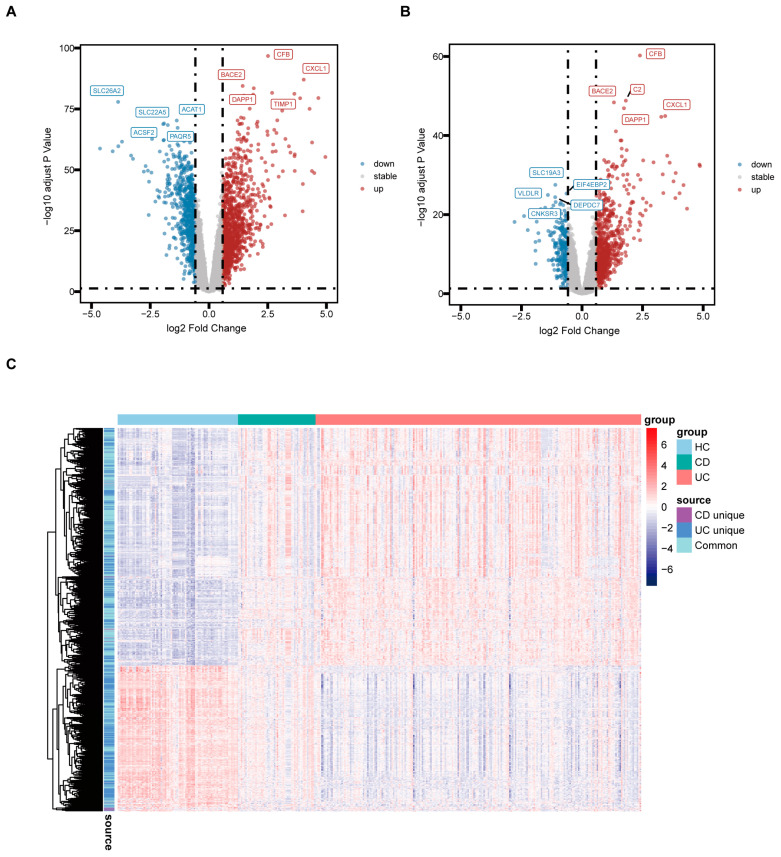
Identification of DEGs associated with UC and CD. (**A**) Volcano plot of DEGs in the colon tissue between UC and HC. (**B**) Volcano plot of DEGs in the colon tissue between CD and HC. (**C**) Heatmap revealing the expression pattern of common and distinct DEGs between groups.

We further analyzed the DEGs according to subtypes of IBD and explicit location. Compared to HC, a total of 1904 DEGs were identified in UC colon tissue, including 1186 up-regulated and 718 down-regulated, while 1037 DEGs were discovered in CD colon tissue, including 761 up-regulated and 276 down-regulated, in line with the thresholds of adjusted *p* < 0.05 and |log2 FC| > 0.58. The volcano plot depicts the top five genes that were up- and down-regulated in UC and CD, respectively (Figure 2A,B). These genes are predominantly known to be associated with the signature of IBD [18,26,47,48]. The summary details of common or exclusive DEGs in UC and CD are provided in Table S3. The heatmap reveals the expression pattern of common and unique DEGs in UC and CD and the relative consistency within the groups (Figure 2C).

### 3.3. Molecular Mechanisms of IBD through GR and TET2-Related Signatures

**Figure 3:**
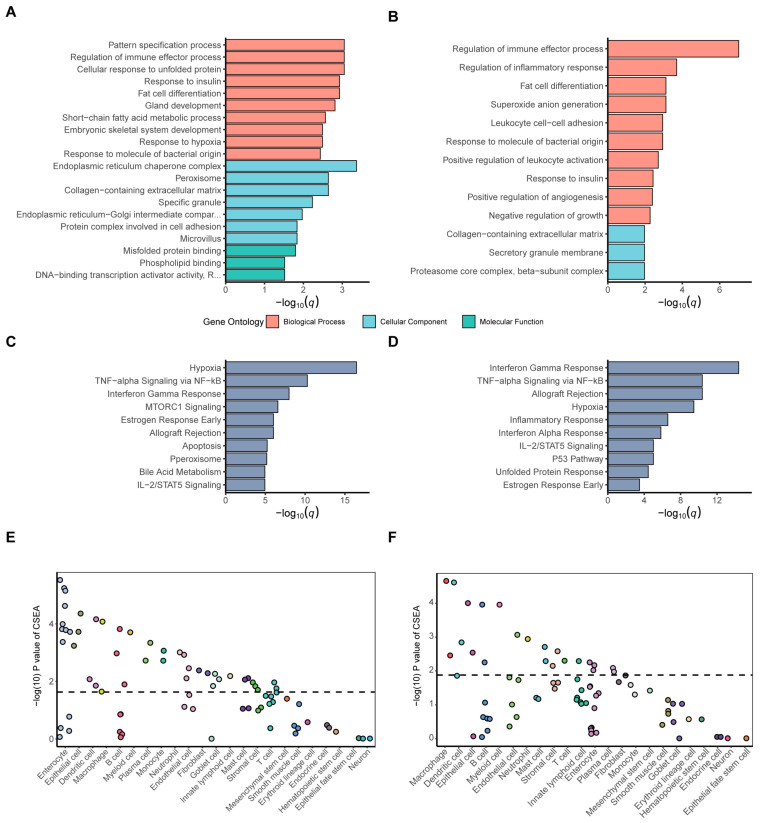
Functional enrichments for IBD signatures around GR and TET2. Top slimmed GO terms for the (**A**) GR- and TET2-related UC DEGs and (**B**) GR- and TET2-related CD DEGs upon the threshold of *q* < 0.05. Top MSigDB hallmark-pathway enrichments for the (**C**) GR- and TET2-related UC DEGs and (**D**) GR- and TET2-related CD DEGs upon the threshold of *q* < 0.05. CSEA testing results of the (**E**) GR- and TET2-related UC DEGs and (**F**) GR- and TET2-related CD DEGs. The *x*-axis indicates the cell types derived from the intestinal tissue and blood. Dots represent the intestine cell types annotated by 23 general classifications descending by order of significance. The dashed line is the significant threshold with FDR < 0.05.

After separately overlapping the DEGs of the two IBD subtypes with GTPCGs, we identified 179 GR- and TET2-related CD DEGs and 401 GR- and TET2-related UC DEGs, as detailed in Tables S4 and S5. We then investigated the potential functions and underlying mechanisms in which they were involved. The top significant slimmed GO enrichment terms (*q* < 0.05) of the respective DEGs are displayed (Figure 3A,B and Table S6). Biological process (BP) of GO analysis illustrated that they were both primarily related to the immune response, such as regulation of immune effector process, response to molecule of bacterial origin, and leukocyte activation and migration. Additionally, response to hypoxia, response to insulin, and multiple metabolic processes were observed. In terms of cellular component (CC) of GO analysis, the terms from GR- and TET2-related UC DEGs were more prevalent than those GR- and TET2-related CD DEGs, and they were particularly associated with endoplasmic reticulum-related components and the peroxisome, except for the collagen-containing extracellular matrix. Concerning molecular function (MF) analysis, the results indicated that misfolded protein binding and DNA-binding transcription activator activity, especially that which was RNA polymerase-II-specific, were the most relevant for the GR- and TET2-related UC DEGs, but the CD DEGs had no significant entries. The GO terms were also consistent with the significant (*q* < 0.05) MSigDB pathways containing hallmark gene sets. The bar charts show that the respective DEGs were both enriched in several commonly activated pathways, including hypoxia, TNF-alpha signaling via NF-κB, interferon gamma response, allograft rejection, and IL-2/STAT5 signaling (Figure 3C,D). In addition to pathways related to inflammatory response and hypoxemic response, fatty acid metabolism, bile acid metabolism, and glycolysis were also observed (Table S7). In summary, according to the results of enrichment analysis, GR- and TET2-related gene signatures are intimately linked with immunometabolism, inflammatory, and cell stress pathways, which may be manifestations of the pathogenesis of IBD.

To further understand which cell type could be responsible for the signatures, we conducted cell-type-specific expression analysis (CSEA) of GR- and TET2-related UC DEGs and CD DEGs. The GR- and TET2-related UC DEGs were most significantly enriched in intestinal enterocytes (FDR = 0.001) among the 23 general cell classifications (Figure 3E and Table S8), while the GR- and TET2-related CD DEGs were predominantly found in macrophages (FDR = 0.003) (Figure 3F and Table S9). The results revealed that the majority of the observed signatures originated from macrophages, dendritic cells, B cells, enterocytes, and epithelial cells, each performing specialized functions in mucosal immunity homeostasis and epithelium barrier function. This implies that changed expression patterns from specific cell subsets might shape disease, in accordance with previous views [2,49,50]. Altogether, the above-mentioned findings suggest that it is potentially valuable to discover novel anti-inflammatory small-molecular drugs for IBD, focusing on transcriptomic signatures associated with GR and TET2.

### 3.4. Establishment and Comparative Transcriptomic Analysis of Two In Vitro Inflammatory Cell Models

Recently, multiple experimental models have been designed to investigate the molecular mechanisms in intestinal inflammation and promote the development of new anti-inflammatory compounds [37,38,39,40,51]. In this study, we utilized two types of intestinal epithelial-derived cells, namely HT29 and HCT116, stimulating them with 1 ug/mL LPS for 24 h to mimic an inflammatory response in the intestine. As shown in Figure S2A, the mRNA level of *NR3C1* was barely detectable in HT29 cells in contrast to HCT116 cells, enabling a comparison of the similarities and differences between these two cell lines with different GR basal expression in response to LPS-induced inflammation. The inflammatory response induced by LPS is known to activate the TLR4/MyD88/NF-κB signaling pathway, which in turn triggers the release of inflammatory cytokines and effector molecules [37,38,52]. We conducted qRT-PCR analysis to determine the expression of TLR4, IL-6, and TNF-α in LPS-stimulated HT29 and HCT116 cells (Figure S2B,C). The results revealed that the mRNA levels of IL-6 and TNF-α were significantly up-regulated in both cell lines after 24 h of induction. Regarding TLR4, there was an up-regulation trend in HCT116 cells compared to the control group, while it was significantly up-regulated in HT29 cells.

**Figure 4:**
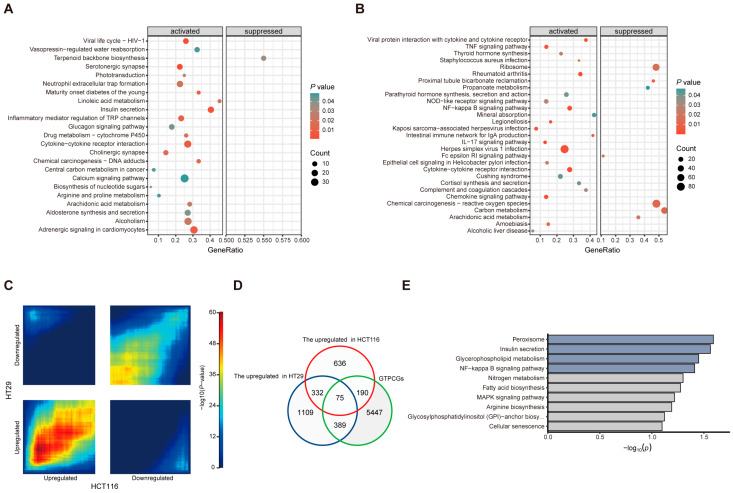
Comparative transcriptome analyses of two in vitro inflammatory cell models. GSEA revealing the overrepresented pathways in overall gene expression of (**A**) HCT116 cells and (**B**) HT29 cells after LPS stimulation. (**C**) RRHO analysis results for comparing expression patterns across the two cell models. (**D**) Venn diagram showing the overlapping genes between specific three gene sets. (**E**) KEGG pathway enrichments for the overlapping genes.

We next performed next-generation RNA sequencing to thoroughly examine the gene expression programs of our in vitro inflammatory models and to identify specific expression signatures useful for screening anti-inflammatory compounds. Gene set enrichment analysis (GSEA) unveiled that immune-response-related signaling pathways were activated in both cell lines following LPS stimulation (Figure 4A,B). Interestingly, HT29 cells showed a more extensive activation of inflammatory-related pathways compared to HCT116 cells in addition to a higher presence of inhibitory pathways. Specifically, Figure 4A highlights the activation of pathways such as the cytokine–cytokine receptor interaction and inflammatory mediator regulation of TRP channels in HT29 cells. In contrast, Figure 4B illustrates the activation of the TNF signaling pathway, NF-κB signaling pathway, intestinal immune network for IgA production, IL-17 signaling pathway, and chemokine signaling pathway in HCT116 cells, with both cell lines sharing the activation of the cytokine–cytokine receptor interaction pathway. Furthermore, RRHO analysis was applied to uncover differential expression (DE) patterns across two cell lines stimulated by LPS. The heatmap displays top-right and bottom-left quadrants, representing overlap in genes down-regulated and up-regulated in both cell lines, while the top-left and bottom-right quadrants represent overlap in genes with oppositional expression patterns between the two cell lines (Figure 4C). Notably, global transcriptomic changes were largely concordant between HT29 and HCT116 cells post LPS induction. By intersecting GTPCGs with gene sets up-regulated in LPS-stimulated HCT116 and HT29 cells, we identified a specific gene signature of 75 genes, indicative of predominantly activated expression programs intensely linked to the LPS-induced effect (Figure 4D). These genes were significantly enriched in the peroxisome, insulin secretion, and NF-κB signaling pathway, which was contained in the enrichment results of the previous GR- and TET2-related gene signatures (Figure 4E). Collectively, our findings indicate that LPS stimulation has an impact on both HCT116 cells with high GR expression and HT29 cells with low GR expression, with the HT29 inflammatory response exhibiting a more pronounced effect. Herein, we selected the inflammation-sensitive HT29 cells as a validation model for the identification of potential anti-inflammatory compounds.

### 3.5. Screening and Validation of Candidate Small-Molecule Compounds for IBD Treatment

**Figure 5:**
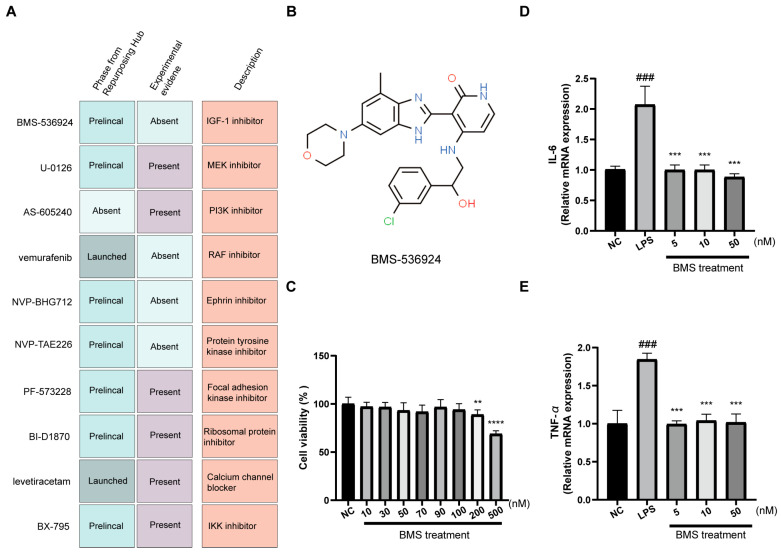
Identification and validation of potential therapeutic compounds. (**A**) The description of top 10 promising small molecules for IBD treatment. (**B**) The chemical structure of the top-ranked BMS-536924. (**C**) The cytotoxicity of BMS-536924 on HT29 cells by CCK8 assay. The effects of BMS-536924 treatment on the expression of proinflammatory cytokines (**D**) IL-6 and (**E**) TNF-α in LPS-induced HT29 cells. Values are expressed as the means ± SD; *n* = 4 in each group. ### *p* < 0.001 vs. the NC group. ** *p* < 0.01, *** *p* < 0.001, and **** *p* < 0.0001 vs. the LPS group.

Based on the above results, CMap analysis was performed to screen small-molecular candidates that might exert a therapeutic effect on IBD, focusing on specific gene signatures associated with GR and TET2. In this regard, the 179 GR- and TET2-related CD DEGs (Table S3), the top 300 GR- and TET2-related UC DEGs (Table S4), and the 75 genes derived from the in vitro inflammation model (Table S10) were employed as transcriptional profiles to query CMap. The top 10 compounds with potential for reversing the inflammatory effect are presented in Figure 5A and Table S11. Remarkably, among the top 10 compounds, six candidates, namely U-0126, AS-605240, PF-573228, BI-D1870, levetiracetam, and BX-795, were previously reported to suppress inflammatory response in mouse colitis models or in vitro inflammatory cell models, which substantiates the reliability of our screening process. Since BMS-536924 emerged as the top-ranked commercially available compound from the CMap analysis, and there was no relevant experimental evidence demonstrating its anti-inflammatory effect, we prioritized it as a candidate small molecule for further validation in the inflammation-sensitive HT29 model. The chemical structure of BMS-536924 is displayed in Figure 5B. The results of the CCK8 assay confirmed that BMS-536924 was not cytotoxic to HT29 cells within the 0–100 nM range (Figure 5C). We next pretreated HT29 cells with LPS and exposed them to the compound at three lower concentrations. The results, illustrated in Figure 5D,E, showed a significant increase in the mRNA levels of the inflammatory cytokines IL-6 and TNF-α due to LPS treatment. However, these levels were markedly reduced following the application of BMS-536924 at each of the three tested concentrations. This outcome demonstrates that BMS-536924 effectively counteracts the upsurge of inflammatory factors induced by LPS, showcasing its potential as a viable therapeutic agent for IBD treatment. The ability of BMS-536924 to significantly reduce the mRNA levels of key inflammatory cytokines, even at lower concentrations, highlights its efficacy in mitigating inflammation, thereby underscoring its potential utility in the management of IBD.

## 4. Discussion

Recent studies increasingly highlight a complex, reciprocal relationship between the glucocorticoid receptor (GR), a widely expressed nuclear factor, and epigenetic enzymes, including HDAC1, DNMT3B, and TET2. This interaction is pivotal in regulating the expression of specific genes in particular biological contexts [14,17,53]. Notably, GR and MAFB have been observed to interact with TET2 in tolerogenic dendritic cells (tolDCs) [17], and our group has further demonstrated the interaction between GR and TET2 through co-immunoprecipitation (Co-IP) and co-localization assays in HEK293T cells (unpublished data). Meanwhile, we performed a one-way ANOVA analysis to examine the differential impact of transcription factor regulation (GR only, TET2 only, and Common) on gene expression levels from public data and observed that the fold-change of the co-regulate genes is markedly different from those regulated by either GR or TET2 alone (Figure S3). The roles of GR and TET2 in regulating immunity and inflammation have been well established independently [9,13]. Therefore, we hypothesize that target gene pathways co-regulated by GR and TET2 are highly correlated with inflammatory responses, which may implicate the pathogenesis of inflammatory diseases such as IBD (including UC and CD) from a new perspective and provide access to potential therapeutic opportunities. In the present study, we conducted comprehensive bioinformatics analyses around GR and TET2 regulation as well as established in vitro inflammatory models with different baseline levels of GR expression, ultimately identifying BMS-536924 as a potential small-molecule drug for IBD.

We identified 179 GR- and TET2-related DEGs in CD and 401 in UC, followed by functional enrichment analyses. The combined results of the MSigDB pathway enrichments with GO terms revealed that these gene signatures around GR and TET2 are involved in the pathogenesis of IBD. Hallmark pathways encompassing inflammatory response, TNF-alpha signaling via NF-κB, IL-6/JAK/STAT3 signaling, hypoxia, peroxisome, and glycolysis were enriched in these gene signatures. This finding supports a previous report that during inflammation of the intestinal mucosa in IBD, superoxide and reactive oxygen intermediates act as antimicrobial agents and also induce oxidative stress, hypoxia, and HIF-1a activation, which in turn induces a metabolic shift towards glycolysis and initiates angiogenesis [50,54]. KEGG pathway enrichment analysis of the specific gene signature derived from the in vitro inflammatory models also showed enrichment in the peroxisome and NF-κB signaling pathway. Previous research has demonstrated that proinflammatory pathways, especially NF-κB, IL-6/STAT3, COX-2/PGE2, and IL-23/Th17, are instrumental in tumorigenesis by triggering the production of inflammatory mediators, elevating the expression of antiapoptotic genes, and stimulating cell proliferation as well as angiogenesis [55]. As a result, IBD patients face a heightened risk of developing colorectal cancer (CRC). This study observed the presence of NF-κB and IL-6/STAT3 pathways among these proinflammatory pathways, underscoring their potential contribution to the increased CRC risk in IBD patients.

The GSEA results of HT29 cells treated with LPS for 24 h uncovered the activation of multiple pathways, including the TNF signaling pathway, NOD-like receptor signaling pathway, NF-κB signaling pathway, intestinal immune network for IgA production, IL-17 signaling pathway, cytokine–cytokine receptor interaction, chemokine signaling pathway, and complement and coagulation cascades. This finding aligns with previous studies indicating that commonly up-regulated pathways in the pathogenesis of IBD (both CD and UC) include the toll-like receptor pathway, NOD-like receptor signaling pathway, cytokine–cytokine interaction, chemokine signaling, intestinal immune network for IgA production, complement and coagulation cascade, and cell adhesion molecules [24,26,47]. This consistency suggests that our in vitro inflammatory model using LPS-induced HT29 cells effectively mirrors the disease state of IBD. Additionally, when compared to HCT116 cells, which have high GR expression, HT29 cells with minimal GR expression showed an intensified inflammatory response to LPS, as evidenced by their overall transcriptome profiles. This disparity at the cellular level complements recent research utilizing a mouse model of DSS-induced colitis. That study confirmed that the absence of intestinal epithelial GR exacerbated clinical symptoms and tissue damage and compromised epithelial barrier integrity during colitis [12]. This cell-level transcriptomic analysis offers valuable insights and supports the understanding of GR’s role in modulating inflammatory responses in IBD.

Specific gene signatures associated with GR and TET2 derived from UC, CD, and the in vitro inflammatory model were further leveraged to screen for small-molecule compounds capable of reversing the disease effect. To the best of our knowledge, this study is the first to identify and validate the anti-inflammatory properties of BMS-536924 in an in vitro inflammatory cell model, highlighting its potential as a therapeutic agent for IBD. BMS-536924 was initially recognized in a 2005 study as a novel inhibitor targeting the insulin-like growth factor receptor (IGF-1R) kinase and the insulin receptor (IR), demonstrating antitumor activity both in vitro and in vivo [56]. More recently, BMS-536924 was shown to effectively reduce the viability of both TMZ-sensitive and -resistant glioblastoma cells and significantly inhibit glioma tumor growth in vivo [57]. This compound primarily exerts its anti-neoplastic effects by inhibiting IGF-1R signaling, which impacts key pathways such as the Ras/Raf/MAPK pathway, which is crucial for cell growth and proliferation, and the PI3K/Akt/mTOR pathway, known for its roles in anti-apoptosis functions and metabolic processes [56,58]. Interestingly, our MSigDB pathway enrichment results also highlighted these pathways, specifically KRAS signaling up and mTORC1 signaling. This suggests that the anti-inflammatory action of BMS-536924 might be linked to its influence on these pathways, providing a novel perspective on its mechanism of action and potential applicability in treating IBD. In addition, GSK4529, also an IGF-1R inhibitor, has been reported to have anti-inflammatory effects in the diabetic kidney mouse model, which provides an important guideline for our future research [59].

A few shortcomings of this research warrant recognition. Firstly, the integrated microarray analysis to identify UC and CD DEGs involved different cohorts with varying sample sizes, which could introduce heterogeneity and potentially obscure some biological differences. Nevertheless, we successfully identified transcriptomic signatures predominantly associated with UC and CD and coinciding with the disease pathogenesis, as previously described. Secondly, the gene expression from bulk microarray data limited the refined comparison of molecular mechanisms related to the major IBD subtypes UC and CD. A more granular approach, such as single-cell analysis, might provide deeper insights into these complex diseases. Moreover, while the CMap offers a systematic approach to gauge the similarity of small-molecule-induced transcriptional changes to normal or altered physiologic states by correlating the gene expression signatures associated with each, it inherently carries the uncertainty of extrapolating expression patterns from cell lines or animal models to human systems. The anti-inflammatory effect of BMS-536924 requires further in vivo validation, such as using a mouse model of colitis, and the latent anti-inflammatory mechanisms warrant in-depth investigation prior to clinical utilization of the compound for treating IBD.

## 5. Conclusions

In this study, we highlighted the significance of transcriptomic signatures associated with GR and TET2 in IBD (UC and CD) and compared the in vitro inflammatory models of two intestinal epithelial cells. Specific gene signatures of disease effects in vivo and in vitro were leveraged to identify the potential IBD-therapeutic small molecule BMS-536924, which may also prevent the onset of CRC. These findings shed light on the pathogenesis of IBD from a new perspective and provide a paradigm for screening small-molecule drugs to reverse the specific disease effect based on gene expression signatures.

## Figures and Tables

**Table 1:** Primers used for qRT-PCR in this study.

Gene	Forward Primer Sequence (5′-3′)	Reverse Primer Sequence (5′-3′)	Product Size
GAPDH	ACCTGACCTGCCGTCTAGAA	GGTGTCGCTGTTGAAGTCAGA	140
TLR4	GGCATCTTCAATGGCTTGTCC	AGAGGTCCAGGAAGGTCAAGT	118
TNF-α	CAACCTCCTCTCTGCCATCAAG	GATAGTCGGGCCGATTGATCTC	152
IL-6	TGCAATAACCACCCCTGACC	ATTTGCCGAAGAGCCCTCAG	150
NR3C1	GTGGAAGGACAGCACAATTACC	CCTGTAGTGGCCTGCTGAAT	173

## Data Availability

The datasets presented in this study can be found in online repositories. The names of the repository/repositories and accession number(s) can be found in the article/Supplementary Materials. All the high-throughput sequencing data have been uploaded to the Gene Expression Omnibus (GEO) and are accessible through the GEO SuperSeries accession number GSE250063. All source codes used in the bioinformatics analysis are available in the Supplementary Materials.

## Supplementary Materials

The following supporting information can be downloaded at: https://www.mdpi.com/article/10.3390/biology13020082/s1, Figure S1: Data preprocessing; Figure S2: Construction of the in vitro inflammatory cell models; Figure S3: A one-way ANOVA analysis was performed to examine the differential impact of transcription factor regulation (GR only, TET2 only, and Common) on gene expression levels. Table S1: Characteristics of seven transcriptome datasets used in the study; Table S2: GR and TET2 putative co-target genes (*n* = 6101); Table S3: IBD signatures obtained from microarray analysis; Table S4: 179 GR- and TET2-related CD DEGs; Table S5: 401 GR- and TET2-related UC DEGs; Table S6: GO results after rrvgo of GR- and TET2-related CD and UC DEGs; Table S7: MSigDB pathway results of GR- and TET2-related CD and UC DEGs; Table S8: Cell-type-specific expression analysis (CSEA) on GR- and TET2-related CD DEGs after filtering; Table S9: Cell-type-specific expression analysis (CSEA) on GR- and TET2-related UC DEGs after filtering; Table S10: The genes profile derived from the in vitro inflammation model for CMap querying; Table S11: The top 10 potential compounds for IBD treatment based on CMap analysis; R scripts.docx: All bioinformatics analysis codes.
