# Supplementary material for: Drug Repositioning of Inflammatory Bowel Disease Based on Co-Target Gene Expression Signature of Glucocorticoid Receptor and TET2
Source: Biology (Basel). 2024 Jan 29;13(2):82. doi: 10.3390/biology13020082 (PMC10886832; doi:10.3390/biology13020082)
Supplement: Supplementary file 1 [file biology-13-00082-s001.zip › R scripts.pdf]

```
### integrate seven datasets
rm(list=ls())
library(dplyr)
setwd("…")
array1 <- read.table("./step1_outcome/GSE75214_filtered.txt",sep="\t",header = T, row.names = 1, quote = "",dec = ".")
array2 <- read.table("./step1_outcome/GSE16879_filtered.txt",sep="\t",header = T, row.names = 1, quote = "",dec=".")
array3 <- read.table("./step1_outcome/GSE179285_filtered.txt",sep="\t",header = T, row.names = 1,quote = "",dec = ".")
array4 <- read.table("./step1_outcome/GSE36807_filtered.txt",sep="\t",header = T, row.names = 1,quote = "",dec = ".")
array5 <- read.table("./step1_outcome/GSE73661_filtered.txt",sep="\t",header = T, row.names = 1,quote = "",dec = ".")
array6 <- read.table("./step1_outcome/GSE9452_filtered.txt",sep="\t",header = T, row.names = 1, quote = "",dec = ".")
array7 <- read.table("./step1_outcome/GSE13367_filtered.txt",sep="\t",header = T, row.names = 1,quote = "",dec = ".")
pheno_array1 <- read.table("./step1_outcome/GSE75214_group_list.txt",sep = "\t",header = T,quote = "",dec = ".")
pheno_array2 <- read.table("./step1_outcome/GSE16879_group_list.txt",sep = "\t",header = T,quote = "",dec = ".")
pheno_array3 <- read.table("./step1_outcome/GSE179285_group_list.txt",sep = "\t",header = T,quote = "",dec = ".")
pheno_array4 <- read.table("./step1_outcome/GSE36807_group_list.txt",sep = "\t",header = T,quote = "",dec = ".")
pheno_array5 <- read.table("./step1_outcome/GSE73661_group_list.txt",sep = "\t",header = T,quote = "",dec = ".")
pheno_array6 <- read.table("./step1_outcome/GSE9452_group_list.txt",sep = "\t",header = T,quote = "",dec=".")
pheno_array7 <- read.table("./step1_outcome/GSE13367_group_list.txt",sep = "\t",header = T,quote = "",dec = ".")
pheno_array1 <- pheno_array1 %>%
                select(geo_accession,group) %>%
                mutate(data="GSE75214",country="Belgium")
pheno_array2 <- pheno_array2 %>%
                select(geo_accession,group) %>%
                mutate(data="GSE16879",country="Belgium")
pheno_array3 <- pheno_array3 %>%
                select(geo_accession,group) %>%
                mutate(data="GSE179285",country="USA")
pheno_array4 <- pheno_array4 %>%
                select(geo_accession,group) %>%
                mutate(data="GSE36807",country="UK")
pheno_array5 <- pheno_array5 %>%
                select(geo_accession,group) %>%
                mutate(data="GSE73661",country="Belgium")
pheno_array6 <- pheno_array6 %>%
                select(geo_accession,group) %>%
                mutate(data="GSE9452",country="Denmark")
pheno_array7 <- pheno_array7 %>%
```

```
                select(geo_accession,group) %>%
                mutate(data="GSE13367",country="Denmark")
pheno_all <-
rbind(pheno_array1,pheno_array2,pheno_array3,pheno_array4,pheno_array5,pheno_array6,pheno_array7)
pheno_all <- pheno_all %>%
            mutate(status=ifelse(str_detect(group,"control"),"Normal","IBD")) %>%
            select(geo_accession,group,status,data,country)
array1 <- as.data.frame(t(array1))
array2 <- as.data.frame(t(array2))
array3 <- as.data.frame(t(array3))
array4 <- as.data.frame(t(array4))
array5 <- as.data.frame(t(array5))
array6 <- as.data.frame(t(array6))
array7 <- as.data.frame(t(array7))
expression_all <- Reduce(intersect,list(colnames(array1),colnames(array2),colnames(array3),colnames(array4),
colnames(array5),colnames(array6),colnames(array7)))
array1 <- array1[,colnames(array1) %in% expression_all]
array2 <- array2[,colnames(array2) %in% expression_all]
array3 <- array3[,colnames(array3) %in% expression_all]
array4 <- array4[,colnames(array4) %in% expression_all]
array5 <- array5[,colnames(array5) %in% expression_all]
array6 <- array7[,colnames(array6) %in% expression_all]
array7 <- array8[,colnames(array7) %in% expression_all]
exp_all <- rbind(array1,array2,array3,array4,array5,array6,array7)
### Figure S1
## PCA plot
library(ggsci)
library(ggplot2)
library(FactoMineR)
library(factoextra)
palette <- pal_npg("nrc",alpha = 0.7)(7)
pheno_all$data <- factor(pheno_all$data)
shape_level <- length(levels(pheno_all[["data"]]))
if (shape_level < 15){
  shapes = (0:shape_level) %% 15
} else{
  shapes = c(0:14,c((15:shape_level) %% 110 + 18))
}
dat.pca <- PCA(exp_all, scale.unit=T,graph = F)
pca_plot_bydata <- fviz_pca_ind(dat.pca, geom.ind = "point",
                                mean.point=F,
                                col.ind = pheno_all$data,
                                palette = palette,
                                #addEllipses = T,
```

```
                                    legend.title = "Datasets") + scale_shape_manual(values = shapes)
pca_plot_bydata
## bactch correction
libraray(sva)
pheno_all <- pheno_all %>%
            mutate(batch = case_when(
            data == "GSE13367" ~ 1,
            data == "GSE16879" ~ 2,
            data == "GSE179285" ~ 3,
            data == "GSE36807" ~ 4,
            data == "GSE73661" ~ 5,
            data == "GSE75214" ~ 6,
            data == "GSE9452" ~ 7
))
pheno_all$batch <- factor(pheno_all$batch)
batch <- pheno_all$batch
pheno_all <- pheno_all %>%
             mutate(info=case_when(
               group=="control" ~ 0,
               group=="case_CD" ~ 1,
               group=="case_UC" ~ 2
             ))
pheno_all$info <- factor(pheno_all$info)
edata <- as.data.frame(t(exp_all))
mod <- model.matrix(~info, data = pheno_all)
combat_edata <- ComBat(dat = edata, batch = batch, mod = mod)
combat_exp_ll <- as.data.frame(t(combat_edata))
## PCA plot
dat.pca2 <- PCA(combat_exp_ll, scale.unit=T,graph = F)
pca_plot_bydata2 <- fviz_pca_ind(dat.pca2, geom.ind = "point",
                          mean.point=F,
                          col.ind = pheno_all$data,
                          palette = palette,
                          #addEllipses = T,
                          legend.title = "Datasets")+scale_shape_manual(values = shapes)
pca_plot_bydata2
## heatmap
library(pheatmap)
combat_exp_ll <- as.data.frame(t(combat_exp_ll))
annotation_col <- data.frame(dataset=pheno_all$data,group=pheno_all$group)
annotation_col <- annotation_col %>%
      mutate(group = case_when(
      group == "case_CD" ~ "CD",
      group == "case_UC" ~ "UC",
```

```
          group == "control" ~ "HC",
          TRUE ~ group))
  rownames(annotation_col) <- colnames(combat_exp_ll)
  head(annotation_col)
  ann_colors = list(
      group = c("HC" = "lightskyblue2", "CD" = "lightseagreen", "UC" = "lightcoral"),
      dataset = c("GSE13367" = "#E64B35B2", "GSE16879" = "#4DBBD5B2","GSE179285" =
"#00A087B2","GSE36807" = "#3C5488B2", "GSE73661" = "#F39B7FB2","GSE75214" =
"#8491B4B2","GSE9452" = "#91D1C2B2"))
range(combat_exp_ll)
p <- pheatmap(combat_exp_ll, annotation_col = annotation_col, cluster_rows = T, cluster_cols = T, show_colnames
= F, show_rownames = F, scale = "row", fontsize = 10, fontsize_row = 3, fontsize_col = 3, annotation_colors =
ann_colors, color = colorRampPalette(c("navy","white","red"))(50))
p
# save
save(pheno_all,file = "./step1_outcome/meta.Rdata")
save(exp_all,file = "./step1_outcome/before_combat_exp.Rdata")
save(combat_exp_ll,file="./step1_outcome/combat_expr.Rdata")
save(expression_all,file = "./step1_outcome/overlap_genes.Rdata")

### Figure 1
rm(list=ls())
setwd("…")
library(cowplot)
library(ggplot2)
library(RColorBrewer)
library(dplyr)
load(file = "./step1_outcome/meta.Rdata")
load(file="./step1_outcome/before_combat_exp.Rdata")
load(file="./step1_outcome/combat_expr.Rdata")
load(file = "./step1_outcome/overlap_genes.Rdata")
GR_TET2_cotarget_genes <- read.table("../01_ChIP-seq/GR_and_TET2_cotarget_genes_in_HEK293.txt",header
= F)
IBD_markers <- read.table("./step1_outcome/robust_IBD_markers.txt",header = T,sep = "\t")
IBD_markers <- unique(as.character(IBD_markers$gene))
com_gene <- intersect(IBD_markers,expression_all)
add_gene <- c("NR3C1","TET2")
com_gene <- c(add_gene,com_gene)
met <- select(combat_exp_ll, one_of(com_gene))
met <- met %>%
   mutate(geo_accession=rownames(met)) %>%
   inner_join(pheno_all,by="geo_accession")
met$group <- factor(met$group,levels = c("control","case_CD","case_UC"))
met$status <- factor(met$status,levels = c("Normal","IBD"))
```

```
met$country <- factor(met$country)
## split violin plot
independent_vars <- c("AQP9", "CD27", "HVCN1", "MUC1", "CD40", "PRKAB1", "IL1B", "MMP3", "TIMP1",
                      "CA2", "REG3A", "AIM2", "IFI16", "ICAM1", "VCAM1", "MADCAM1", "TCF4")
select_name <- c(independent_vars,"status")
df <- subset(met,select=select_name)
df_long <- reshape2::melt(df, id.vars = "status", variable.name = "Gene", value.name = "Expression")
# Define a function
if(T){
  GeomSplitViolin <- ggproto("GeomSplitViolin", GeomViolin, draw_group = function(self, data, ...,
draw_quantiles = NULL){
    data <- transform(data, xminv = x - violinwidth * (x - xmin), xmaxv = x + violinwidth * (xmax - x))
    grp <- data[1,'group']
    newdata <- plyr::arrange(transform(data, x = if(grp%%2==1) xminv else xmaxv), if(grp%%2==1) y else -y)
    newdata <- rbind(newdata[1, ], newdata, newdata[nrow(newdata), ], newdata[1, ])
    newdata[c(1,nrow(newdata)-1,nrow(newdata)), 'x'] <- round(newdata[1, 'x'])
    if (length(draw_quantiles) > 0 & !scales::zero_range(range(data$y))) {
      stopifnot(all(draw_quantiles >= 0), all(draw_quantiles <=
                                              1))
      quantiles <- ggplot2:::create_quantile_segment_frame(data, draw_quantiles)
      aesthetics <- data[rep(1, nrow(quantiles)), setdiff(names(data), c("x", "y")), drop = FALSE]
      aesthetics$alpha <- rep(1, nrow(quantiles))
      both <- cbind(quantiles, aesthetics)
      quantile_grob <- GeomPath$draw_panel(both, ...)
      ggplot2:::ggname("geom_split_violin", grid::grobTree(GeomPolygon$draw_panel(newdata, ...),
quantile_grob))
    }
    else {
      ggplot2:::ggname("geom_split_violin", GeomPolygon$draw_panel(newdata, ...))
    }
  })
  geom_split_violin <- function (mapping = NULL, data = NULL, stat = "ydensity", position = "identity", ...,
draw_quantiles = NULL, trim = TRUE, scale = "area", na.rm = FALSE, show.legend = NA, inherit.aes = TRUE) {
    layer(data = data, mapping = mapping, stat = stat, geom = GeomSplitViolin, position = position, show.legend
= show.legend, inherit.aes = inherit.aes, params = list(trim = trim, scale = scale, draw_quantiles = draw_quantiles,
na.rm = na.rm, ...))
  }
}
p2 <- ggplot(df_long, aes(x = Gene, y = Expression, fill = status)) +
      geom_split_violin(trim = T,colour=NA) +
      scale_fill_manual(values = c("lightblue3", "lightcoral")) +
      geom_point(stat = 'summary',fun=mean,
                 position = position_dodge(width = 0.3))+
      stat_summary(fun.min = function(x){quantile(x)[2]},
```

```
                    fun.max = function(x){quantile(x)[4]},
                    geom = 'errorbar',color='black',
                    width=0.01,size=0.5,
                    position = position_dodge(width = 0.3))+
    stat_compare_means(data = df_long,aes(x = Gene, y = Expression), label = "p.signif", method = "t.test") +
    theme_bw() +
    theme(panel.grid.major = element_blank(),panel.grid.minor = element_blank(),axis.line = element_line(colour = "black"),
          legend.position = "top") +
    xlab("")+ ylab("Expression")
p2
```

ROC analysis

```
library(pROC)
library(rms)
df <- df %>%
    mutate(response=ifelse(status=="Normal",0,1))
df$response <- factor(df$response)
ddist <- rms::datadist(df)
options(datadist = 'ddist')
f <- rms::lrm(response ~ PRKAB1 + ICAM1 + CD40 + MUC1, data = df,
              x=T,y=T)
f
OR <- exp(f$coefficients)
OR
f_ROC <- glm(response ~ PRKAB1 + ICAM1 + CD40 + MUC1,data=df,family = binomial())
df$predvalue <- predict(f_ROC,type = "response")
head(df)
ROC <- pROC::roc(df$response,df$predvalue)
round(auc(ROC),3) # AUC 0.985
ci(auc(ROC)) # 95% CI: 0.9745-0.9962
roc_PRKAB1 <- pROC::roc(df$response,df$PRKAB1)
round(auc(roc_PRKAB1),3) # 0.738
ci(auc(roc_PRKAB1)) # 95% CI: 0.6784-0.7967
roc_ICAM1 <- pROC::roc(df$response,df$ICAM1)
round(auc(roc_ICAM1),3) # 0.916
ci(auc(roc_ICAM1)) # 95% CI: 0.8809-0.9502
roc_CD40 <- pROC::roc(df$response,df$CD40)
round(auc(roc_CD40),3) # 0.953
ci(auc(roc_CD40)) # 95% CI: 0.9317-0.974
roc_MUC1 <- pROC::roc(df$response,df$MUC1)
round(auc(roc_MUC1),3) # 0.924
ci(auc(roc_MUC1)) # 95% CI: 0.8975-0.9508
plot(1-ROC$specificities,
     ROC$sensitivities,type="l",
```

```
        col="#E64B35FF",
        lty=1,
        xlab = "1-Specificity",
        ylab = "Sensitivity",
        lwd=2)
lines(1-roc_PRKAB1$specificities,roc_PRKAB1$sensitivities,type="l",col="#F39B7FFF",lty=1,lwd=2)
lines(1-roc_ICAM1$specificities,roc_ICAM1$sensitivities,type="l",col="#3C5488FF",lty=1,lwd=2)
lines(1-roc_CD40$specificities,roc_CD40$sensitivities,type="l",col="#00A087FF",lty=1,lwd=2)
lines(1-roc_MUC1$specificities,roc_MUC1$sensitivities,type="l",col="#4DBBD5FF",lty=1,lwd=2)
abline(0,1,col="darkgrey",lty=4)
legend(0.45,0.3,
       c("CD40-AUC = 0.953","MUC1-AUC = 0.924","ICAM1-AUC = 0.916","PRKAB1-AUC = 0.738",
                    "TotalMarkers-AUC = 0.985"),
                     lty=c(1,1,1,1,1),
                     lwd=c(2,2,2,2,2),
                     col = c("#00A087FF","#4DBBD5FF","#3C5488FF","#F39B7FFF","#E64B35FF"),
                     bty = "o")
## dot plot of correlation analysis
setwd("…")
data <- openxlsx::read.xlsx("cor.test_result.xlsx")
dt <- data %>% select(other_gene,gene,estimate.cor,p.value)
colnames(dt) <- c("Gene","Focus","Correlation","P_Value")
dt$pvalue_group <- cut(dt$P_Value, breaks = c(0, 0.0001, 0.001, 0.01, 0.05,1), labels = c("<0.0001", "<0.001", "<0.01", "<0.05", ">0.05"))
dt$pvalue_group <- factor(dt$pvalue_group, levels = c(labels = c("<0.0001","<0.001", "<0.01", "<0.05", ">0.05")))
dt$pvalue_group_2 <- cut(dt$P_Value, breaks = c(0, 0.0001, 0.001, 0.01, 0.05, 1), labels = c("<0.0001","<0.001", "<0.01", "<0.05", ">0.05"))
dt$pvalue_group_2 <- factor(dt$pvalue_group_2, levels = c(labels = c("<0.0001","<0.001", "<0.01", "<0.05", ">0.05")))
dt <- dt %>% filter(!(Focus == "NR3C1" & dt$Gene == "TET2" | Focus == "TET2" & dt$Gene == "NR3C1"))
ggplot()+
geom_hline(yintercept = dt$Focus,color = "#E8E8E8")+
geom_point(data = subset(dt, Correlation > 0),shape = 19,stroke = 0,
             aes(x = Gene, y = Focus,size = abs(Correlation),color = pvalue_group_2))+
geom_point(data = subset(dt, Correlation < 0),shape = 21,stroke = 0.1,
             aes(x = Gene, y = Focus, size = abs(Correlation),fill = pvalue_group))+
scale_size_continuous(limits = c(0, 0.8),breaks = c(0.1, 0.2, 0.3, 0.4, 0.5, 0.6,0.7,0.8))+
scale_fill_manual(values = c(rev(brewer.pal(9,'YlGnBu'))[3:6],"#E8E8E8"))+
scale_color_manual(values = c(rev(brewer.pal(9,'Reds'))[5:8],"#E8E8E8"))+
theme_cowplot() +
coord_flip() +
theme(panel.grid.major = element_blank(),
        axis.text.x = element_text(angle = 45, hjust = 1),
        panel.border = element_rect(color = "black", fill = NA, linewidth = 1),
```

```
              plot.title = element_text(hjust = 0.5,))+
labs(fill = "Negative\ncorrelation\np value", size = "Pearson's r",
            color = "Positive\ncorrelation\np value",x = "",y = "") +
guides(size = guide_legend(override.aes = list(size = c(2:9), fill = "white"),order = 1),
              color = guide_legend(override.aes = list(size = 4),order = 2),
              fill = guide_legend(override.aes = list(size = 4),order = 3))
## Immune infiltration analysis
library(CIBERSORT)
rm(list=ls())
setwd("…")
load(file = "./step1_outcome/meta.Rdata")
load(file="./step1_outcome/before_combat_exp.Rdata")
load(file="./step1_outcome/combat_expr.Rdata")
mixed_expr <- t(combat_exp_ll)
data(LM22)
View(LM22)
results <- cibersort(sig_matrix = LM22, mixture_file = mixed_expr, perm=1000,QN=T)
re <- as.data.frame(results)
dt <- merge(re,pheno_all,by.x="row.names",by.y="geo_accession")
colnames(dt)[1] <- "sample"
dt <- dt %>% dplyr::mutate("group" = dplyr::recode_factor(`group`,
                                                          case_CD = "CD",
                                                          case_UC = "UC",
                                                          control = "HC"))
table(dt$group,dt$status)
dt <- dt %>% dplyr::select(sample,group,status,everything())
dt <- dt[,1:25]
dt_long <- dt %>% tidyr::pivot_longer(cols = colnames(.)[4:25],names_to = "cell.type",values_to = "value")
library(ggplot2)
library(ggsci)
library(ggpubr)
library(ggforce)
library(viridis)
dt_long$status <- factor(dt_long$status,levels = c("Normal","IBD"))
dt_long$group <- factor(dt_long$group,levels = c("HC","CD","UC"))
p1 <- ggplot(dt_long,aes(cell.type,value,fill = status)) +
          geom_boxplot(outlier.shape = 21,color = "black") +
  theme_bw() +
  scale_fill_manual(values = c("cadetblue3","darksalmon")) +
  labs(x = "", y = "Estimated proportion") +
  theme(legend.position = "top",axis.line = element_line(colour = "black"),axis.text.x = element_text(angle=80,vjust = 0.5)) +
  stat_compare_means(aes(group = status),label = "p.signif",method = "t.test")
p1
```

```
# according to the boxplot with t-test to select significantly different celltypes for subquent analysis
sig_cell <- c("Dendritic cells activated","Macrophages M0","Macrophages M1","Macrophages M2",
              "Mast cells activated","Mast cells resting","Neutrophils","NK cells activated","NK cells resting",
              "Plasma cells","T cells CD4 memory activated","T cells CD4 naive","T cells CD8","T cells
               follicular helper", "T cells regulatory (Tregs)")
df <- dplyr::select(dt,one_of(sig_cell))
rownames(df) <- dt$sample
key_gene <- c("NR3C1","TET2","MUC1", "CD40", "PRKAB1", "ICAM1")
met <- dplyr::select(combat_exp_ll, one_of(key_gene))
df <- merge(df,met,by.x = "row.names",by.y = "row.names")
rownames(df) <- df$Row.names
df <- df[,-1]
result_list <- list()
for (gene in key_gene) {
  cor_results <- apply(df[,1:15], 2, function(x) {
    test_result <- cor.test(df[,gene], x, method="spearman")
    return(c(estimate = test_result$estimate, p.value = test_result$p.value))
  })
  cor_df <- as.data.frame(t(cor_results))
  cor_df$gene <- gene
  cor_df$cell_types <- rownames(cor_df)
  result_list[[gene]] <- cor_df
}
final_result <- do.call(rbind, result_list)
cor_result <- final_result %>%
  dplyr::mutate("correlation significance"=ifelse(p.value<0.05,"significant","ns"))%>%
  dplyr::select(gene,cell_types,"correlation significance",everything())
cor_result <- cor_result[!grepl("NR3C1|TET2",cor_result$gene),]
library(cowplot)
library(RColorBrewer)
data <- cor_result %>% dplyr::select(cell_types,gene,estimate.rho,p.value)
colnames(data) <- c("Cell","Focus","Correlation","P_Value")
data$pvalue_group <- cut(data$P_Value, breaks = c(0, 0.0001, 0.001, 0.01, 0.05,1), labels = c("<0.0001", "<0.001",
"<0.01", "<0.05", ">0.05"))
data$pvalue_group <- factor(data$pvalue_group, levels = c(labels = c("<0.0001","<0.001", "<0.01", "<0.05",
">0.05")))
data$pvalue_group_2 <- cut(data$P_Value, breaks = c(0, 0.0001, 0.001, 0.01, 0.05, 1), labels =
c("<0.0001","<0.001", "<0.01", "<0.05", ">0.05"))
data$pvalue_group_2 <- factor(data$pvalue_group_2, levels = c(labels = c("<0.0001","<0.001", "<0.01", "<0.05",
">0.05")))
ggplot2::ggplot()+
  geom_hline(yintercept = data$Focus,color = "#E8E8E8")+
  geom_point(data = subset(data, Correlation > 0),shape = 19,stroke = 0,
```

```
                    aes(x = Cell, y = Focus,size = abs(Correlation),color = pvalue_group_2))+
    geom_point(data = subset(data, Correlation < 0),shape = 21,stroke = 0.1,
                    aes(x = Cell, y = Focus, size = abs(Correlation),fill = pvalue_group))+
    scale_size_continuous(limits = c(0, 0.8),breaks = c(0.1, 0.2, 0.3, 0.4, 0.5, 0.6,0.7,0.8))+
    scale_fill_manual(values = c(rev(brewer.pal(9,'YlGnBu'))[3:6],"#E8E8E8"))+
    scale_color_manual(values = c(rev(brewer.pal(9,'Reds'))[5:8],"#E8E8E8"))+
    cowplot::theme_cowplot() +
    coord_flip() +
    theme(panel.grid.major = element_blank(),
          axis.text.x = element_text(angle = 45, hjust = 1),
          panel.border = element_rect(color = "black", fill = NA, linewidth = 1),
          plot.title = element_text(hjust = 0.5,))+
    labs(fill = "Negative\ncorrelation\np value", size = "Spearman's p",
         color = "Positive\ncorrelation\np value",x = "",y = "") +
    guides(size = guide_legend(override.aes = list(size = c(2:9), fill = "white"),order = 1),
           color = guide_legend(override.aes = list(size = 4),order = 2),
           fill = guide_legend(override.aes = list(size = 4),order = 3))
```

differential expression analysis

```
library(limma)
library(dplyr)
design <- model.matrix(~0+group,data=pheno_all)
contrast.matrix <- makeContrasts("groupcase_CD-groupcontrol","groupcase_UC-groupcontrol", levels=design)
contrast.matrix
combat_exp_ll <- as.data.frame(t(combat_exp_ll))
fit <- lmFit(combat_exp_ll,design)
fit2 <- contrasts.fit(fit, contrast.matrix)
fit2 <- eBayes(fit2)
tempOutput1 <- topTable(fit2, coef=1, n=Inf)
tempOutput2 <- topTable(fit2, coef=2, n=Inf)
nrDEG1 <- na.omit(tempOutput1)
nrDEG2 <- na.omit(tempOutput2)
nrDEG1$`-log10(adj.P.Val)` <- -log10(nrDEG1$adj.P.Val)
nrDEG2$`-log10(adj.P.Val)` <- -log10(nrDEG2$adj.P.Val)
nrow(subset(nrDEG1,adj.P.Val<0.05)) # 5958
nrow(subset(nrDEG2,adj.P.Val<0.05)) # 8919
logFC=0.58
adj.P.Val=0.05
type1 <- (nrDEG1$logFC < -logFC)&(nrDEG1$adj.P.Val < adj.P.Val)
type2 <- (nrDEG1$logFC > logFC)&(nrDEG1$adj.P.Val < adj.P.Val)
nrDEG1$change <- ifelse(type1,"down",ifelse(type2,"up","stable"))
table(nrDEG1$change) # CD: down 276 up 761
type3 <- (nrDEG2$logFC < -logFC)&(nrDEG2$adj.P.Val < adj.P.Val)
type4 <- (nrDEG2$logFC > logFC)&(nrDEG2$adj.P.Val < adj.P.Val)
```

```
nrDEG2$change <- ifelse(type3,"down",ifelse(type4,"up","stable"))
table(nrDEG2$change) # UC: down 718 up 1186
output1 <- nrDEG1 %>%
  filter(abs(logFC)>0.58,adj.P.Val<0.05)
output2 <- nrDEG2 %>%
  filter(abs(logFC)>0.58,adj.P.Val<0.05)
# write
write.table(output1,file = "CD_degs.xlsx",row.names = T,col.names = T,sep = "\t")
write.table(output2,file = "UC_degs.xlsx",row.names = T,col.names = T,sep = "\t")
### Figure 2
## Volcano plot
library(ggplot2)
library(ggrepel)
library(ggprism)
p1 <- ggplot(nrDEG1, aes(x =logFC, y=-log10(adj.P.Val), color=change)) +
  geom_point(alpha=0.65, size=1.5) +
  scale_color_manual(values=c('steelblue','gray','brown'),limits = c('down', 'stable', 'up')) +
  xlim(c(-5, 5)) +
  geom_vline(xintercept=c(-logFC, logFC),lty=4,col="black",lwd=0.8)+
  geom_hline(yintercept = -log10(adj.P.Val), lty=4,col="black",lwd=0.8) +
  labs(x = 'log2 Fold Change', y = '-log10 adjust P Value') +
  theme(plot.title = element_text(hjust = 0.5),legend.position="right",
        legend.title = element_blank()
        )+
  ggprism::theme_prism(border = T)
volcano_plot1 <- p1 + ggrepel::geom_label_repel(data = label1,aes(label=rownames(label1)),
                                                size=3, box.padding = unit(0.5,"lines"),
                                                point.padding = unit(0.8,"lines"),
                                                segment.color = "black", show.legend = F,
max.overlaps = 10000)
volcano_plot1
p2 <- ggplot(nrDEG2, aes(x =logFC, y=-log10(adj.P.Val), color=change)) +
  geom_point(alpha=0.65, size=1.5) +
  scale_color_manual(values=c('steelblue','gray','brown'),limits = c('down', 'stable', 'up')) +
  xlim(c(-5, 5)) +
  geom_vline(xintercept=c(-logFC, logFC),lty=4,col="black",lwd=0.8)+
  geom_hline(yintercept = -log10(adj.P.Val), lty=4,col="black",lwd=0.8) +
  labs(x = 'log2 Fold Change', y = '-log10 adjust P Value') +
  theme(plot.title = element_text(hjust = 0.5),legend.position="right",
        legend.title = element_blank()
  )+
  ggprism::theme_prism(border = T)
volcano_plot2 <- p2 + ggrepel::geom_label_repel(data = label2,aes(label=rownames(label2)),
                                                size=3, box.padding = unit(0.5,"lines"),
```

```r
                                                            point.padding = unit(0.8,"lines"),
                                                            segment.color = "black", show.legend = F,
max.overlaps = 10000)
volcano_plot2
## heatmap
library(pheatmap)
library(dplyr)
CD_UC_common <- intersect(CD_degs$gene,UC_degs$gene)
CD_uniq <- setdiff(CD_degs$gene,CD_UC_common)
UC_uniq <- setdiff(UC_degs$gene,CD_UC_common)
merge_IBD <- union(CD_degs$gene,UC_degs$gene)
combat_exp_ll <- as.data.frame(t(combat_exp_ll))
diff_merge <- combat_exp_ll[which(rownames(combat_exp_ll) %in% merge_IBD),]
new_df <- data.frame(Gene = merge_IBD, Source = "")
new_df$Source[new_df$Gene %in% CD_UC_common] <- "Common"
new_df$Source[new_df$Gene %in% CD_uniq] <- "CD unique"
new_df$Source[new_df$Gene %in% UC_uniq] <- "UC unique"
head(new_df)
diff_merge <- diff_merge %>%
  mutate(Gene=rownames(diff_merge)) %>%
  inner_join(new_df,by="Gene")
diff_merge <- diff_merge %>%
  select(Gene,Source,everything())
annotation_col <- data.frame(group=pheno_all$group,geo=pheno_all$geo_accession)
annotation_col <- annotation_col %>%
  mutate(group = case_when(
    group == "case_CD" ~ "CD",
    group == "case_UC" ~ "UC",
    group == "control" ~ "HC",
    TRUE ~ group
  )) %>%
  arrange(factor(group, levels = c("HC", "CD", "UC")))
diff <- diff_merge %>% select(-c(Gene,Source))
rownames(diff) <- diff_merge$Gene
diff_t <- as.data.frame(t(diff))
diff <- merge(annotation_col,diff_t,by.x = "geo",by.y = "row.names")
diff <- diff %>% arrange(factor(group,levels = c("HC", "CD", "UC")))
rownames(diff) <- diff$geo
diff <- diff[,-c(1,2)]
diff_t <- as.data.frame(t(diff))
diff <- diff_t
rm(diff_t)
rownames(annotation_col) <- colnames(diff)
head(annotation_col)
```

```
annotation_col <- annotation_col[,1]
annotation_col <- as.data.frame(annotation_col)
rownames(annotation_col) <- colnames(diff)
colnames(annotation_col) <- "group"
annotation_row <- data.frame(source=diff_merge$Source)
rownames(annotation_row) <- rownames(diff)
head(annotation_row)
ann_colors = list(
  group = c("HC" = "lightskyblue2", "CD" = "lightseagreen", "UC" = "lightcoral"),
  source = c("CD unique" = "#BA55D3", "UC unique" = "#6495ED","Common"="#B0E0E6")
)
range(diff)   # -1.815732 14.995396
p <- pheatmap(diff,annotation_col = annotation_col,annotation_row = annotation_row, cluster_rows = T,
cluster_cols = F,
         show_colnames = F,show_rownames = F, scale = "row",fontsize = 10,fontsize_row = 3,
         fontsize_col = 3,annotation_colors = ann_colors,color = colorRampPalette(c("navy","white","red"))(50))
p

### Figure 3
setwd("…")
library(ggpubr)
library(ggplot2)
library(clusterProfiler)
library(org.Hs.eg.db)
library(stats)
library(data.table)
library(dplyr)
CD_degs <- openxlsx::read.xlsx("./step1_outcome/CD_degs.xlsx")
UC_degs <- openxlsx::read.xlsx("./step1_outcome/UC_degs.xlsx")
cotarget <- read.table("../01_ChIP-seq/GR_and_TET2_cotarget_genes_in_HEK293.txt",header = F)
length(intersect(UC_degs$gene,cotarget$V1)) # 401 UC_cotargets
length(intersect(CD_degs$gene,cotarget$V1)) # 179 CD_cotargets
UC_cotarget_CSEA <- UC_degs[which(UC_degs$gene %in% intersect(UC_degs$gene,cotarget$V1)),]
CD_cotarget_CSEA <- CD_degs[which(CD_degs$gene %in% intersect(CD_degs$gene,cotarget$V1)),]
## functional analysis
geneG1.df <- bitr(UC_cotarget_CSEA$gene, fromType = "SYMBOL",
                  toType = c("ENTREZID", "SYMBOL"),
                  OrgDb = org.Hs.eg.db)
colnames(geneG1.df)[1] <- "gene"
head(geneG1.df)
UC_data <- left_join(geneG1.df,UC_cotarget_CSEA,by="gene")
UC_GO <- enrichGO(gene = UC_data$ENTREZID,
                  keyType = "ENTREZID",
                  OrgDb=org.Hs.eg.db,
```

```
                        ont = "ALL",
                        pvalueCutoff = 0.05,
                        pAdjustMethod = "BH",
                        minGSSize = 10,
                        maxGSSize = 500,
                        qvalueCutoff = 0.05,
                        readable = TRUE)
UC_GOresult <- data.frame(UC_GO)

geneG2.df <- bitr(CD_cotarget_CSEA$gene, fromType = "SYMBOL",
                        toType = c("ENTREZID", "SYMBOL"),
                        OrgDb = org.Hs.eg.db)
colnames(geneG2.df)[1] <- "gene"
head(geneG2.df)
CD_data <- left_join(geneG2.df,CD_cotarget_CSEA,by="gene")
CD_GO <- enrichGO(gene = CD_data$ENTREZID,
                        keyType = "ENTREZID",
                        OrgDb=org.Hs.eg.db,
                        ont = "ALL",
                        pvalueCutoff = 0.05,
                        pAdjustMethod = "BH",
                        minGSSize = 10,
                        maxGSSize = 500,
                        qvalueCutoff = 0.05,
                        readable = TRUE)
CD_GOresult <- data.frame(CD_GO)

library(enrichR)
dbs <- listEnrichrDbs()
dbs                                                                                                   <-
c("MSigDB_Hallmark_2020","GO_Biological_Process_2023","GO_Cellular_Component_2023","GO_Molecular
_Function_2023")
UC_enrichr <- enrichr(geneG1.df$gene, dbs)
CD_enrichr <- enrichr(geneG2.df$gene, dbs)
setwd("…")
results <- list(CD = CD_enrichr,UC = UC_enrichr)
purrr::walk(names(results), function(contrast) {
  print(glue::glue("Analyzing enrichR for {contrast}"))
  results[[contrast]] %>%
  purrr::set_names(names(.) %>% stringr::str_trunc(31, ellipsis = "")) %T>%
  openxlsx::write.xlsx(file = glue::glue("enrichr_{contrast}.xlsx"))
# reduce redundant GO
library(rrvgo)
for (name in c("UC_GOresult","CD_GOresult")) {
```

```
  dt <- get(name)
  merged_rrvgo_result <- data.frame()
  types <- unique(dt$ONTOLOGY)
  for (var in types){
    go_analysis <- dt[dt$ONTOLOGY==var,]
    simMatrix <- calculateSimMatrix(go_analysis$ID,
                                    orgdb="org.Hs.eg.db",
                                    ont=var,
                                    method="Rel")
    scores <- setNames(-log10(go_analysis$qvalue), go_analysis$ID)
    reducedTerms <- reduceSimMatrix(simMatrix,
                                    scores,
                                    threshold=0.7,
                                    orgdb="org.Hs.eg.db")
    result <- go_analysis[which(go_analysis$ID %in% unique(reducedTerms$parent)),]
    merged_rrvgo_result <- rbind(merged_rrvgo_result,result)
  }
  openxlsx::write.xlsx(merged_rrvgo_result,file = paste0(name,"_after_rrvgo.xlsx"))
}
# production plot
GOplot <- function(contrast = contrast){
  openxlsx::read.xlsx(glue::glue("…/enrichment_analysis/{contrast}_GOresult_after_rrvgo.xlsx")) %>%
    dplyr::mutate("Gene Ontology" = dplyr::recode_factor(`ONTOLOGY`,
                                                         BP = "GO Biological Process",
                                                         CC = "GO Cellular Component",
                                                         MF = "GO Molecular Function")) %>%
    dplyr::select(-`ONTOLOGY`) %>%
    dplyr::mutate(Description = stringr::str_trim(Description)) %>%
    dplyr::mutate(Description = Hmisc::capitalize(Description)) %>%
    dplyr::mutate(Description = stringr::str_trunc(Description, 50)) %>%
    dplyr::group_by(`Gene Ontology`) %>%
    dplyr::slice_head(n=10) %>%
    dplyr::ungroup() %>%
    dplyr::mutate(Description = factor(.$Description, levels = unique(.$Description[order(forcats::fct_rev(.$`Gene
Ontology`), .$`-log10.qvalue`)]))) %>%
    ggplot2::ggplot(aes(x = Description,
                        y = `-log10.qvalue`,
                        fill = `Gene Ontology`,
                        group = `Gene Ontology`)) +
    ggplot2::geom_bar(stat = "identity",
                      position = position_dodge(),
                      color = "Black",
                      size = 0.25) +
    ggplot2::coord_flip() +
```

```
      ggplot2::scale_y_continuous(expand = c(0, 0, 0.1, 0)) +
      ggsci::scale_fill_npg("nrc",alpha = 0.6) +
      #ggsci::scale_fill_ds() +
      ggplot2::labs(y = expression("-log"[10](italic(q)))) +
      ggplot2::theme_classic() +
      ggplot2::theme(text = element_text(size = 12),
                     axis.title.y = element_blank(),
                     axis.title.x = element_text(size = 12),
                     plot.title = element_text(face = "bold", size = 12),
                     legend.position = "none") +
      ggtitle(dplyr::case_when(contrast == "CD" ~ "GR/TET2-related CD DEGs",
                               contrast == "UC" ~ "GR/TET2-related UC DEGs")) %>%
      return()
}
pathwayPlot <- function(contrast = contrast){
   readxl::read_xlsx(glue::glue("…/enrichment_analysis/enrichr_{contrast}.xlsx"), sheet = "MSigDB_Hallmark_2020") %>%
      dplyr::mutate("Gene Ontology" = "MSigDB_Hallmark_2020",
                    "-log10.qvalue" = -log10(Adjusted.P.value)) %>%
      dplyr::select("Gene Ontology", Term, "-log10.qvalue", Combined.Score) %>%
      dplyr::mutate(Database = dplyr::recode_factor(`Gene Ontology`, "MSigDB_Hallmark_2020" = "MSigDB Pathways")) %>%
      dplyr::select(-`Gene Ontology`) %>%
      dplyr::mutate(Term = Hmisc::capitalize(Term)) %>%
      dplyr::mutate(Term = stringr::str_trim(Term)) %>%
      dplyr::mutate(Term = stringr::str_trunc(Term, 50)) %>%
      dplyr::slice(1:10) %>%
      dplyr::mutate(Term = factor(.$Term, levels = rev(.$Term))) %>%
      ggplot2::ggplot(aes(x = Term,
                          y = `-log10.qvalue`,
                          fill = Database,
                          group = Database)) +
      ggplot2::geom_bar(stat = "identity",
                        position = position_dodge(),
                        color = "Black",
                        size = 0.25) +
      ggplot2::coord_flip() +
      ggplot2::scale_y_continuous(expand = c(0, 0, 0.1, 0)) +
      ggplot2::scale_fill_manual(values = "#3C548899") +
      #ggplot2::scale_fill_manual(values = ggsci::pal_d3()(4)[4]) +
      ggplot2::labs(y = expression("-log"[10](italic(q)))) +
      ggplot2::theme_classic() +
      ggplot2::theme(text = element_text(size = 12),
                     axis.title.y = element_blank(),
```

```
                              axis.title.x = element_text(size = 12),
                              plot.title = element_text(face = "bold", size = 12),
                              #legend.position = "bottom")+
                              legend.position = "none") +
    ggtitle("MSigDB Pathways") %>%
    return()
}

GOplot("UC")
GOplot("CD")
pathwayPlot("UC")
pathwayPlot("CD")
cowplot::plot_grid(GOplot("UC"),
                              GOplot("CD"),
                              pathwayPlot("UC"),
                              pathwayPlot("CD"),
                              ncol = 2,
                              align = "v",
                              labels = c("A", "B", "C","D"),
                              label_size = 12,
                              rel_heights = c(1.6,1),
                              scale = 0.95) %>%
   ggplot2::ggsave("GRTET2-related DEGs functional enrichments.pdf",
                             plot = .,
                             width = 12,
                             height = 8)
```

CSEA plot

```
rm(list=ls())
library(randomcoloR)
library(ggplot2)
library(dplyr)
setwd("…")
cell_enrich=read.table("./WebCSEA/UC_cotarget/WebCSEA_All_tissue_cell_type.txt",header = T,sep = "\t")
cell_enrich$FDR=p.adjust(cell_enrich$input_list_combined_p,method = "BH")
select=data.frame(table(cell_enrich$General_cell_type))
select=select[select$Freq>=10,]
cell_enrich=cell_enrich[cell_enrich$General_cell_type %in% select$Var1,]
cell_enrich=cell_enrich[cell_enrich$Tissue %like% "Intestine" | cell_enrich$Tissue %like% "Colon",]
orders=as.character(cell_enrich$General_cell_typ[!duplicated(cell_enrich$General_cell_typ)])
cell_enrich$General_cell_type=factor(cell_enrich$General_cell_type,levels = orders)
View(cell_enrich)
cell_enrich <- subset(cell_enrich,select = -delabel)
length(orders) # 23
```

```
n <- 23
palette <- distinctColorPalette(n)
ggplot(cell_enrich,aes(General_cell_type, input_list_combined_log10p)) +
  geom_jitter(aes(fill=General_cell_type), shape =21,size=4)+
  guides(fill=F)+
  theme_bw()+
  theme(panel.grid.major = element_blank(),
        panel.grid.minor = element_blank(), axis.line = element_line(colour = "black"))+
  geom_hline(yintercept=1.63,linetype="dashed", color = "black")+
  theme(axis.text.x = element_text(angle = 45, vjust = 1, hjust=1))+
  xlab("")+ylab("-log(10) P value of CSEA")+
  scale_fill_manual(values = palette)
csea_genes <- read.table("./WebCSEA/UC_cotarget/WebCSEA_all_gene_list_result.tsv",header = F,sep = "\t")
names(csea_genes) <- c("Tissue_cell_type_name","Genes")
met1 <- merge(cell_enrich,csea_genes,by.x = "Tissue_cell_type_name",by.y = "Tissue_cell_type_name")
str(met1)
met1 <- met1[order(met1$input_list_combined_log10p,decreasing = T),]
openxlsx::write.xlsx(met1,file = "… /CSEA on UC after filtering.xlsx")

cell_enrich=read.table("./WebCSEA/CD_cotarget/WebCSEA_All_tissue_cell_type.txt",header = T,sep = "\t")
cell_enrich$FDR=p.adjust(cell_enrich$input_list_combined_p,method = "BH")
select=data.frame(table(cell_enrich$General_cell_type))
select=select[select$Freq>=10,]
cell_enrich=cell_enrich[cell_enrich$General_cell_type %in% select$Var1,]
cell_enrich=cell_enrich[cell_enrich$Tissue %like% "Intestine" | cell_enrich$Tissue %like% "Colon",]
orders=as.character(cell_enrich$General_cell_typ[!duplicated(cell_enrich$General_cell_typ)])
cell_enrich$General_cell_type=factor(cell_enrich$General_cell_type,levels = orders)
cell_enrich <- subset(cell_enrich,select = -delabel)
length(orders) # 23
n <- 23
palette <- distinctColorPalette(n)
View(cell_enrich)
ggplot(cell_enrich,aes(General_cell_type, input_list_combined_log10p)) +
  geom_jitter(aes(fill=General_cell_type), shape =21,size=4)+
  guides(fill=F)+theme_bw()+
  theme(panel.grid.major = element_blank(),
        panel.grid.minor = element_blank(), axis.line = element_line(colour = "black"))+
  geom_hline(yintercept=1.88,linetype="dashed", color = "black")+
  theme(axis.text.x = element_text(angle = 45, vjust = 1, hjust=1))+xlab("")+ylab("-log(10) P value of
CSEA")+scale_fill_manual(values = palette)
csea_genes <- read.table("./WebCSEA/CD_cotarget/WebCSEA_all_gene_list_result.tsv",header = F,sep = "\t")
names(csea_genes) <- c("Tissue_cell_type_name","Genes")
met2 <- merge(cell_enrich,csea_genes,by.x = "Tissue_cell_type_name",by.y = "Tissue_cell_type_name")
met2 <- met2[order(met2$input_list_combined_log10p,decreasing = T),]
```

```
openxlsx::write.xlsx(met2,file = "…/CSEA on CD after filtering.xlsx")
}

### Figure 4
## GSEA
setwd("…")
load(file = "./HCT116_14960genes_dt.Rdata")
load(file = "./HT29_13508genes_dt.Rdata")
packages <- c("clusterProfiler","fgsea","org.Hs.eg.db","GSEABase","dplyr","tidyverse","msigdbr","stringr","ggplot2","enrichplot","DOSE","pathview","topGO")
stopifnot(suppressMessages(sapply(packages,require,character.only=T)))
options(clusterProfiler.download.method = "wininet")
names(result1)[1] <- "SYMBOL"
rt1 <- bitr(geneID = result1$SYMBOL,fromType = "SYMBOL",
            toType = "ENTREZID",OrgDb = org.Hs.eg.db)
DEG <- merge(result1,rt1,by = "SYMBOL")
DEG <- DEG %>%
       select(ENTREZID,logFC,SYMBOL)
genelist1 <- DEG$logFC
names(genelist1) <- as.character(DEG$ENTREZID)
head(genelist1)
genelist1 <- sort(genelist1, decreasing = TRUE)
gsea1 <- gseKEGG(geneList = genelist1,
                 organism = 'hsa',
                 keyType = "kegg",
                 exponent = 1,
                 minGSSize = 10,
                 maxGSSize = 500,
                 pvalueCutoff = 0.05,
                 pAdjustMethod = "none",
                 by = "fgsea")
g1v <- setReadable(gsea1,'org.Hs.eg.db','ENTREZID')
View(g1v@result)
write.table(g1v@result,file=paste0(".../all_GSEA_HCT116-1.xls"),sep="\t",quote=F,col.names=T,row.names = F)

names(result2)[1] <- "SYMBOL"
rt2 <- bitr(geneID = result2$SYMBOL,fromType = "SYMBOL",
            toType = "ENTREZID",OrgDb = org.Hs.eg.db)
DEG <- merge(result2,rt2,by = "SYMBOL")
DEG <- DEG %>%
       select(ENTREZID,logFC,SYMBOL)
genelist2 <- DEG$logFC
names(genelist2) <- as.character(DEG$ENTREZID)
```

```
head(genelist2)
genelist2 <- sort(genelist2, decreasing = TRUE)
gsea2 <- gseKEGG(geneList = genelist2,
                 organism = 'hsa',
                 keyType = "kegg",
                 exponent = 1,
                 minGSSize = 10,
                 maxGSSize = 500,
                 pvalueCutoff = 0.05,
                 pAdjustMethod = "none",
                 by = "fgsea")
g2v <- setReadable(gsea2,'org.Hs.eg.db','ENTREZID')
View(g2v@result)
write.table(g2v@result,file=paste0(".../all_GSEA_HT29-1.xls"),sep="\t",quote=F,col.names=T,row.names = F)
g1 <- g1v@result
g2 <- g2v@result
num1 <- g1[,c(1,11)]
num1 <- num1 %>%
  separate_rows(core_enrichment, sep = "/") %>%
  group_by(ID)%>%
  count()
num1 <- num1[match(g1$ID,num1$ID),]
sum(num1$ID==g1$ID)
g1$Count <- num1$n
data1 <- g1 %>% mutate(GeneRatio = Count/setSize)
data1$sign <- ifelse(data1$NES>0,"activated","suppressed")
num2 <- g2[,c(1,11)]
num2 <- num2 %>%
  separate_rows(core_enrichment, sep = "/") %>%
  group_by(ID)%>%
  count()
num2 <- num2[match(g2$ID,num2$ID),]
sum(num2$ID==g2$ID)
g2$Count <- num2$n
data2 <- g2 %>% mutate(GeneRatio = Count/setSize)
data2$sign <- ifelse(data2$NES>0,"activated","suppressed")
p1 <- ggplot(data1)+
  geom_point(aes(x=GeneRatio, y=Description, size=Count, color=pvalue))+
  facet_grid(~sign,scales = "free") +
  scale_colour_gradient(low = "#E64B35FF",high = "cadetblue")+
  labs(color="P value", size="Count") +
  guides(color=guide_colourbar(order = 1),size=guide_legend(order = 2)) +
  theme_bw()+
  theme(axis.title.y = element_blank())
```

```
p1
p2 <- ggplot(data2)+
  geom_point(aes(x=GeneRatio, y=Description, size=Count, color=pvalue))+
  facet_grid(~sign,scales = "free") +
  scale_colour_gradient(low = "#E64B35FF",high = "cadetblue")+
  labs(color="P value", size="Count") +
  theme_bw()+
  theme(axis.title.y = element_blank())
p2
```

RRHO analysis

```
rm(list=ls())
setwd("…")
load(file = "./HCT116_14960genes_dt.Rdata")
load(file = "./HT29_13508genes_dt.Rdata")
library(RRHO2)
library(dplyr)
inputdata1 <- data.frame(
  genes = as.character(result1$genes),
  input_score = -log10(result1$PValue) * ifelse(result1$logFC > 0, 1, -1)
)
inputdata2 <- data.frame(
  genes = as.character(result2$genes),
  input_score = -log10(result2$PValue) * ifelse(result2$logFC > 0, 1, -1)
)
common_genes <- intersect(inputdata1$genes, inputdata2$genes) # 12434
inputdata1 <- inputdata1[inputdata1$genes %in% common_genes, ]
inputdata2 <- inputdata2[inputdata2$genes %in% common_genes, ]
# Make sure the gene symbols are sorted in the same order in both data frames
inputdata1 <- inputdata1[order(inputdata1$genes), ]
inputdata2 <- inputdata2[order(inputdata2$genes), ]
# Identify non-unique gene identifiers in inputdata1
print(inputdata1[duplicated(inputdata1$genes), ]$genes)
# Remove duplicate genes from inputdata1
inputdata1 <- inputdata1[!duplicated(inputdata1$genes), ]

print(inputdata2[duplicated(inputdata2$genes), ]$genes)
inputdata2 <- inputdata2[!duplicated(inputdata2$genes), ]
common_genes <- intersect(inputdata1$genes, inputdata2$genes)
inputdata1 <- inputdata1[inputdata1$genes %in% common_genes, ]
inputdata2 <- inputdata2[inputdata2$genes %in% common_genes, ]
# Initialize the RRHO object
RRHO_obj <- RRHO2_initialize(inputdata1, inputdata2, labels = c("HCT116 LPS vs NC", "HT29 LPS vs NC"),
log10.ind = TRUE)
# Create the heatmap
```

```
RRHO2_heatmap(RRHO_obj)
uu <- RRHO_obj$genelist_uu
GR_TET2_cotarget_genes <- read.table("…/GR_and_TET2_cotarget_genes_in_HEK293.txt",header = F)
df <- list("up-genes HT29"=uu$gene_list2_uu,"up-genes HCT116"=uu$gene_list1_uu,"GTPCGs"=GR_TET2_cotarget_genes$V1)
# Venn
library(ggVennDiagram)
library(ggsci)
library(ggplot2)
ggVennDiagram(df, label_alpha=0,label_size = 4,label = "count", edge_size = 0.5) +
  scale_color_lancet() +
  scale_fill_gradient(low="gray99",high = "gray95",guide="none")
## KEGG
rm(list=ls())
setwd("…")
uu_GRTET <- readr::read_delim("…/RROH2_genelist_output/uu_GR_TET2.txt",delim = "\t") #75 obs
library(clusterProfiler)
library(org.Hs.eg.db)
names(uu_GRTET)[1] <- "SYMBOL"
rt1 <- bitr(geneID = uu_GRTET$SYMBOL,fromType = "SYMBOL",
            toType = "ENTREZID",OrgDb = org.Hs.eg.db)
options(clusterProfiler.download.method = "wininet")
kegg1 <- enrichKEGG(rt1$ENTREZID, organism = 'human', pvalueCutoff = 1,
                    pAdjustMethod = 'none', minGSSize = 10, maxGSSize = 500, qvalueCutoff = 1,
                    use_internal_data = F)
result_kegg1 <- data.frame(DOSE::setReadable(kegg1,'org.Hs.eg.db','ENTREZID'))
result_kegg1 <- result_kegg1 %>%
   dplyr::mutate(Sig=ifelse(`pvalue`<0.05,"significant","no")) %>%
   dplyr::mutate("-log10.pvalue"=-log10(pvalue)) %>%
   dplyr::select(Description,"-log10.pvalue",Sig,geneID) %>%
   dplyr::mutate(Description = Hmisc::capitalize(Description)) %>%
   dplyr::mutate(Description = stringr::str_remove(Description, "R-HSA.*$")) %>%
   dplyr::mutate(Description = stringr::str_trim(Description)) %>%
   dplyr::mutate(Description = stringr::str_trunc(Description, 50)) %>%
   dplyr::slice(1:10) %>%
   dplyr::mutate(Description = factor(.$Description, levels = rev(.$Description)))
ggplot2::ggplot(result_kegg1,aes(x = Description,y = `-log10.pvalue`,fill = Sig,group = Sig)) +
  ggplot2::geom_bar(stat = "identity",
                    position = position_dodge(),
                    color = "Black",
                    size = 0.25) +
  ggplot2::coord_flip() +
  ggplot2::scale_y_continuous(expand = c(0, 0, 0.1, 0)) +
  ggplot2::scale_fill_manual(values = c("#3C548899","gray80"),limits=c("significant","no")) +
```

```
  ggplot2::labs(y = expression("-log"[10](italic(p)))) +
  ggplot2::theme_classic() +
  ggplot2::theme(text = element_text(size = 12),
                 axis.title.y = element_blank(),
                 axis.title.x = element_text(size = 12),
                 plot.title = element_text(face = "bold", size = 12),
                 legend.position = "none")
```

### a one-way ANOVA analysis to examine the differential impact of transcription factor regulation (GR only, TET2 only, and Common) on gene expression levels

```
rm(list=ls())
setwd("…")
load(file="…/combat_expr.Rdata")
load(file = "…/overlap_genes.Rdata")
GR_putative_target_genes <- read.table("HEK293_NR3C1_GSM1176699_41735.txt",header = F,sep = "\t",quote = "",dec=".")
GR_putative_target_genes <- data.frame(gene_id=GR_putative_target_genes$V1,tf="GR")
GR_putative_target_genes <- GR_putative_target_genes[which(GR_putative_target_genes$gene_id %in% expression_all),]
TET2_putative_target_genes <- read.table("HEK293T_TET2_putative_target_genes.txt",header = F)
TET2_putative_target_genes <- data.frame(gene_id=TET2_putative_target_genes$V1,tf="TET2")
TET2_putative_target_genes <- TET2_putative_target_genes[which(TET2_putative_target_genes$gene_id %in% expression_all),]
cotarget_genes <- data.frame(gene_id=intersect(GR_putative_target_genes$gene_id,TET2_putative_target_genes$gene_id),tf="Common")
GR_target_genes_only <- data.frame(gene_id=setdiff(GR_putative_target_genes$gene_id,cotarget_genes$gene_id),tf="GR_only")
TET2_target_genes_only <- data.frame(gene_id=setdiff(TET2_putative_target_genes$gene_id,cotarget_genes$gene_id),tf="TET2_only")
gene_info <- rbind(cotarget_genes,GR_target_genes_only,TET2_target_genes_only)

combat_exp_ll$gene_id <- rownames(combat_exp_ll)
combat_exp_ll <- combat_exp_ll %>% dplyr::filter(!gene_id %in% c("NR3C1","TET2"))
combat_exp_long <- tidyr::pivot_longer(combat_exp_ll, cols = -gene_id, names_to = "sample", values_to = "expression")
combat_exp_long_dt <- data.table::as.data.table(combat_exp_long)
gene_info_dt <- data.table::as.data.table(gene_info)
data.table::setkey(combat_exp_long_dt, gene_id)
data.table::setkey(gene_info_dt, gene_id)
combined_data <- data.table::merge.data.table(combat_exp_long_dt, gene_info_dt, by = "gene_id")
combined_data$tf <- factor(combined_data$tf)
anova_result <- aov(expression ~ tf, data = combined_data)
summary(anova_result) # p < 2e-16 ***
```

```
library(ggplot2)
ggplot(combined_data, aes(x = tf, y = expression, fill = tf)) +
  geom_boxplot(outlier.shape = 21,color="black") +
  ggsci::scale_fill_npg("nrc",alpha = 0.8)+
  ggpubr::stat_compare_means(method = "anova") +
  theme_bw() +
  labs(x = "", y = "Expression Level")
```
