# Supplementary material for: Drug Repositioning of Inflammatory Bowel Disease Based on Co-Target Gene Expression Signature of Glucocorticoid Receptor and TET2
Source: Biology (Basel). 2024 Jan 29;13(2):82. doi: 10.3390/biology13020082 (PMC10886832; doi:10.3390/biology13020082)
Supplement: Supplementary file 1 [file biology-13-00082-s001.zip › Supplementary Figures.pdf]

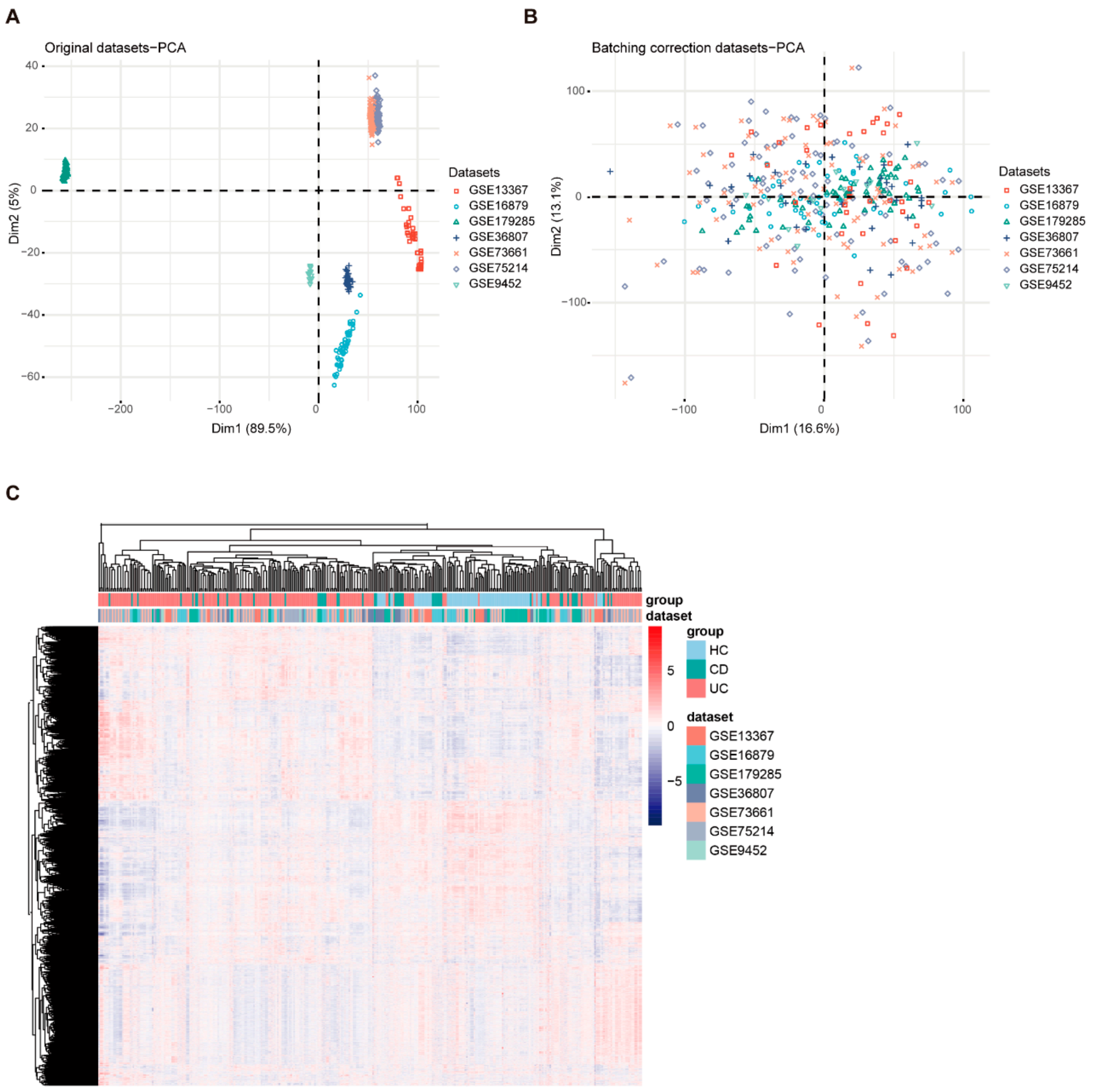

Figure S1. Data preprocessing. (**A**) PCA of original IBD datasets before bath-effect correction. (**B**) PCA of the integrated dataset after batch-effect correction. (C) A heatmap of the integrated dataset after batch-effect.

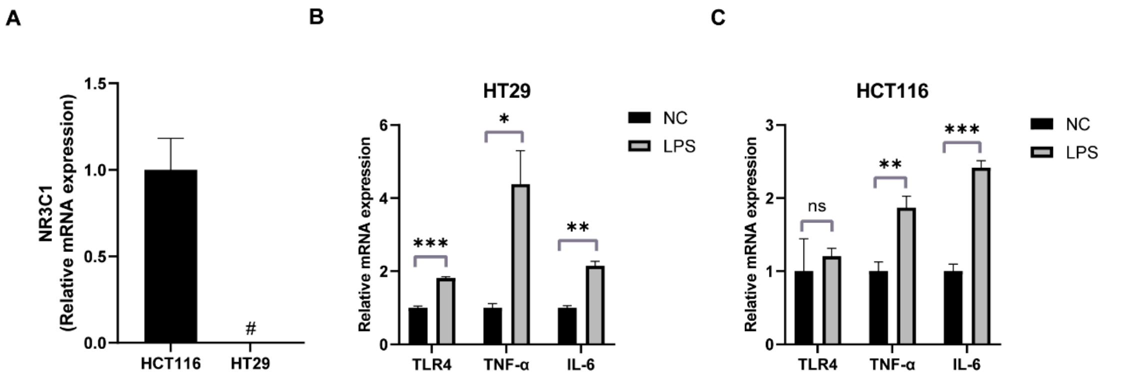

Figure S2. Construction of the *in vitro* inflammatory cell models. (**A**) Comparison of the baseline levels of GR expression between HT29 cells and HCT116 cells. # bare expression compared to the HCT116 cells. The expression of inflammatory factors TLR4, TNF-α, and IL-6 in (**B**) HT29 cells

and (**C**) HCT116 cells both treated with LPS for 24 h. Values are expressed as the means ± SD, n = 3 in each group. * $p < 0.05$, ** $p < 0.01$, *** $p < 0.001$, and ns not significant.

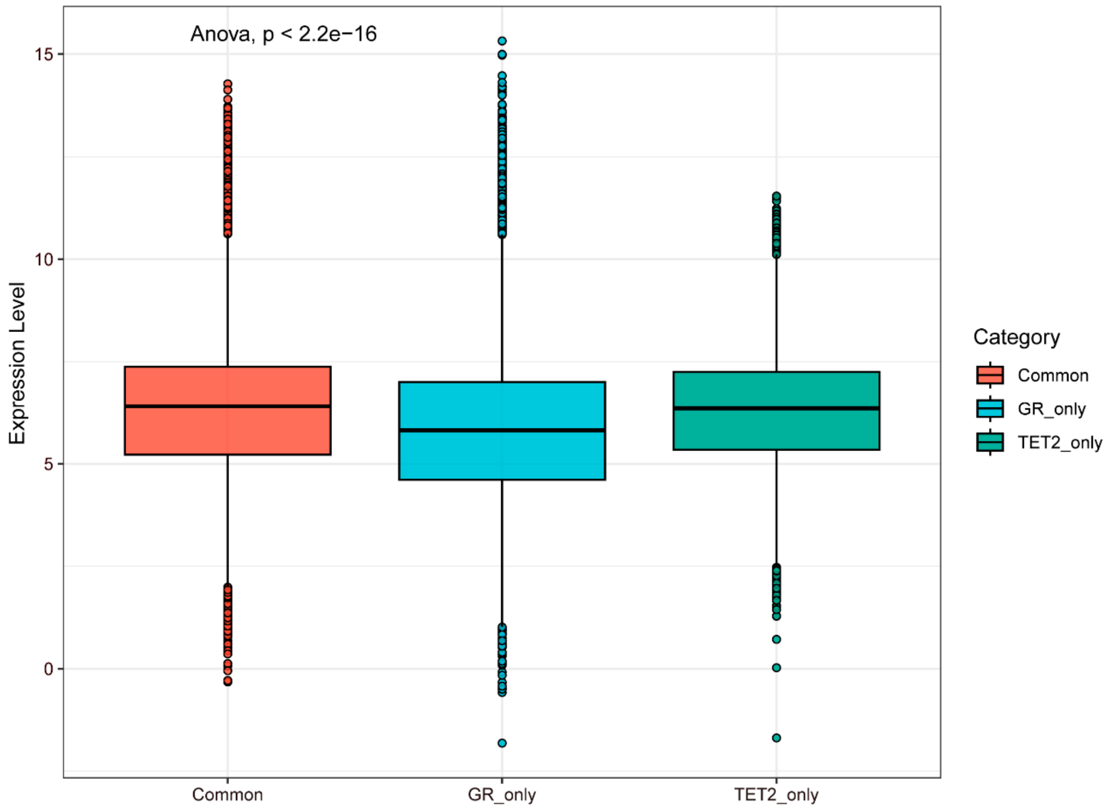

Figure S3. A one-way ANOVA analysis was performed to examine the differential impact of transcription factor regulation (GR only, TET2 only, and Common) on gene expression levels.
